# BNT162b2 mRNA vaccination affects the gut microbiome composition of patients with follicular lymphoma and chronic lymphocytic leukemia

**DOI:** 10.1186/s40364-025-00734-w

**Published:** 2025-02-10

**Authors:** Annalisa Chiarenza, Gaia Vertillo Aluisio, Nunziatina Laura Parrinello, Sara Marino, Anna Maria Corsale, Grete Francesca Privitera, MojtabaShekarkar Azgomi, Enrico La Spina, Daniela Cambria, Angelo Curtopelle, Gaetano Isola, Cirino Botta, Francesco Di Raimondo, Alessandra Romano, Maria Santagati

**Affiliations:** 1 Divisione Di Ematologia, AOU Policlinico Rodolico San Marco, Catania, Italy; 2 Department of Biomedical and Biotechnological Sciences (BIOMETEC), Section Microbiology University of Catania, Catania, Italy; 3 Dipartimento Di Chirurgia E Specialità Medico Chirurgiche, Università Degli Studi Di Catania, Catania, Italy; 4 Department of Health Promotion, Mother and Child Care, Internal Medicine and Medical Specialties, University of Palermo, Palermo, Italy; 5 Department of Clinical and Experimental Medicine, BioinformaticsUnit, University of Catania, Catania, Italy

**Keywords:** Chronic lymphatic leukemia, Follicular lymphoma, COVID-19, Vaccination, Myeloid composition, Immunome, Microbiome profiling

## Abstract

**Background:**

In both chronic lymphatic leukemia (CLL) and follicular lymphoma (FL) immunotherapy determines B-depletion that leads to temporary suppression of humoral immunity, which is clinically relevant especially during the COVID-19 pandemic, when most patients in the first wave received the BNT162b2 vaccine during anti-neoplastic treatment.

**Methods:**

To capture changes in the immunome and microbiome composition in CLL and FL patients upon mRNA-based vaccination, we designed a prospective, longitudinal study to profile both the humoral and the cellular response after exposure to the BNT162b2 COVID-19 vaccine.

**Results:**

In both CLL patients and FL patients, the second and third administrations of the BNT162b2 vaccine increased the titer of specific antibodies against SARS-CoV-2. In FL patients, vaccination induced expansion of central memory CD8 + CD57dim CD279 + T cells and reduction of the neutrophil subset myeloid 1 (CD14^−^CD15^+^CD16^dim^CD64^+^CD33^−^CD38^+^PDL1^+^HLA-DR^−^); in both cohorts, CD45RA + CD27 + CD279 + NK cells were expanded after a full cycle of vaccination. After vaccination, the genera Collinsella, Gemmiger, Lachnospiraceae, Blautia, Ruminococcus and Lactobacillus increased in both CLL patients and FL patients, whereas Faecalibacterium, Enterobacteriacae, and Enterococcus decreased. Multivariate analysis failed to identify factors associated with changes in microbiome communities among the CLL and FL cohorts, considering age, sex, exposure to anti-CD20 therapy and disease activity. Only in FL patients, alpha diversity was negatively correlated with neutrophil subsets myeloid 1 e 5 at baseline and positively correlated with neutrophil subset 6 after vaccination. PICRUSt2 analysis showed how microbiome can also affect the host health promoting chronic inflammation. The L-lysine biosynthesis pathway was more represented in CLL patients, whereas the L-valine degradation pathway and the anaerobic degradation of purine nucleobases were overrepresented in the FL cohort.

**Conclusions:**

Taken together, our findings reveal the effect of the BNT162b2 vaccine in shaping the microbiome composition in CLL and FL patients, despite receiving treatment for their underlying active disease, and highlight the importance of a comprehensive analysis of the immunome and microbiome profiling to understand immune function in these cohorts of patients.

**Supplementary Information:**

The online version contains supplementary material available at 10.1186/s40364-025-00734-w.

## Introduction

The COVID-19 pandemic has had a profound impact on the management and outcomes of patients with hematologic malignancies, including follicular lymphoma (FL) and chronic lymphocytic leukemia (CLL) [1–4]. In FL and CLL, immune surveillance is impaired due to abnormalities in antigen presentation and T-cell receptor signaling pathways [5]. In CLL, exhausted T cells exhibit increased expression of inhibitory receptors such as PD-1 (programmed cell death protein 1) and CTLA-4 (cytotoxic T- lymphocyte-associated protein 4), which dampen their effector functions and contribute to immune evasion by tumor cells [6].

BNT162b2 is a lipid nanoparticle-encapsulated mRNA vaccine developed to protect against the SARS- CoV-2 virus and has been shown to be highly effective at reducing the risk of severe illness, hospitalization, and death from COVID-19, especially in individuals with a weakened immune system. Due to the increased risk for severe COVID-19 because of both their underlying disease and the immunosuppressive effects of their treatments, FL and CLL patients have been prioritized for BNT162b2 vaccination during pandemics [7, 8]. International practice guidelines suggest completing vaccination 2–4 weeks before starting the anti-CD20 monoclonal antibodies Rituximab and obinutuzumab [9], which are often not practicable in real-life settings for life-threatening underlying lymphoproliferative disorders. In our center, BNT162b2 was offered to all frail patients, including FL and CLL individuals, who were receiving active anti-neoplastic treatment. Starting from the reduced protection against SARS-CoV-2 infection in CLL and FL patients [4, 7], longitudinal studies investigating the T-cell response to multiple-dose vaccination have been conducted to understand the immune function of FL/CLL patients in vivo.

In healthy subjects, the BNT162b2 vaccine decreases the abundance and diversity of the gut microbiome, which could affect the immunogenicity of the BNT162b2 vaccine and its effectiveness. The suboptimal response to the BNT162b2 COVID-19 vaccine in patients with FL and CLL has been largely attributed to defects in the humoral response and immunological impairment of B and T cells [4, 6, 7, 10, 11] but has been poorly investigated in relation to microbiome composition [12]. For this reason, we comprehensively profiled microbiome composition and both the humoral and cellular responses after 1 month from the second dose of the BNT162b2 COVID-19 vaccine in a prospective, longitudinal study, involving FL and CLL patients, to understand the relationship between the immune function and the microbiome composition in FL/CLL patients and identifying potential biomarkers useful for novel immunotherapies and future vaccination strategies.

## Materials and methods

### Patient population and inclusion criteria

The study recruited 38 patients with lymphoproliferative disorders (19 with CLL and 19 with FL) evaluated between 22 April and 6 December 2021. All participants received two doses of vaccine at the same vaccination center (A.O.U. Rodolico San Marco), BNT162b2 mRNA (Comirnaty, Pfizer- BioNTech), 30 mcg per dose, via intramuscular injection three weeks before, as indicated in the Italian national guide. Among these, 15 also received a boost (third) dose after 4 weeks from the second. Despite this, patients who had recently completed or even still underwent anti-CD20 therapy waited approximately 6–9 months for the administration of the vaccine to ensure greater protection and to avoid possible alteration of the therapy. Patients with previous SARS-CoV-2 exposure or vaccination were excluded. All included patients had no concurrent parasitic, bacterial, or viral co-infection and underwent antibiotic and antiviral treatment as standard prophylaxis (800/160 mg of sulfamethoxazole/ trimethoprim bid twice a week and antiviral prophylaxis with Acyclovir 400 mg bid). The main clinical characteristics, including body mass index (BMI), age, gender and blood cell counts, of the subjects included in the study are summarized in Table 1 and Supplementary Table 1.

**Table 1:** Demographics of subjects included in the study

	**Healthy**	**CLL**	**FL**	*p*-value*
**Subjects, n**	40	19	19	
**Age, median (range)**	56 (36–66)	68 (49–86)	60 (42–74)	0.66
**Gender**				
Female, n (%)	26 (65)	7 (37)	6 (32)	0.06
**IgM, mg/dL**				
median (range)	83 (60–130)	45 (6–150)	70 (53–92)	0.91
**ANC, 10**^**3**^**cells/mmc**	4.4	4.1	3.8	0.09
median (range)	(2.8–7.2)	(1.2–9.4)	(1.8–6.3)	
**ALC, 10**^**3**^**cells/mmc**	2.8	2.6	1.3	0.09
median (range)	(1.5–4.3)	(0.5–56.5)	(0.4–2.7)	
**AMC, 10**^**3**^**cells/mmc**	0.6	0.4	0.5	0.99
median (range)	(0.3–0.9)	(0.3–1.2)	(0.2–0.6)	
**Body Mass Index**				
median (range)	25.2 (19.3–33.8)	23.8 (21.1–31.9)	25.4 (19.8–28.3)	0.81

*Abbreviations: FL* follicular lymphoma, *CLL* chronic lymphatic leukemia, *ANC* absolute neutrophil count, *ALC* absolute lymphocyte count, *AMC* absolute monocyte count

^*^ANOVA test

The study was approved by the Institutional Review Board (IRB) (Comitato etico Catania 1, #CO.TIP. 34/2020/PO 0016693 released on April 15th, 2020) and performed according to the principles of the Declaration of Helsinki and the International Conference on Harmonization Good Clinical Practice guidelines.

Following signed consent from the patients, peripheral blood samples were collected at three different timepoints: the first before the first dose (T1), the second 15 days after the second dose (T2) and the last 15 days after the third dose (T3). All the samples were analyzed at the same laboratory via the same procedure: suitable tubes were used for biochemical and hematological analysis; specifically, the tubes containing plasma and heparin were processed within two hours after collection and immediately stored at −20 °C. While these serological analyses were used to compare CLL and FL patients to healthy subjects and their response to vaccination, microbiological analysis was carried out only in 15 patients affected by CLL (*N* = 7, 5 females and 2 males) and FL (*N* = 8, 2 females and 6 males), who were aged between 43 and 79 years. In this subgroup the clinical and immunological findings were further associated with changes in the gut microbiome during vaccination.

### Serological response to vaccination

Serological response to COVID-19 vaccination in CLL and FL patients was compared to that achieved in 40 healthy subjects, while immunome and microbiome composition changes were followed only in CLL/FL patients according to a case–control study design, to reduce bias of selection like diet, basal metabolism, anti-neoplastic treatment. The health records of subjects followed in our hospital were evaluated to capture the following information: details and timing of the tumor treatment and laboratory parameters at the time of the first and second doses of SARS-CoV-2 vaccination, followed by a booster shot (third dose), as previously described [13, 14].

Serum was obtained from 5 ml of peripheral clotted blood after centrifugation at 900 × g and stored in aliquots at − 20 °C. All samples and data were deidentified after collection, and researchers conducting the assays were blinded to the clinical data until the final comparative analysis was performed.

Immunogenicity was assessed via a chemiluminescent microparticle immunoassay (SARS-CoV-2 IgG II Quant assay on an ALINITY analyzer; Abbott) to quantify IgG antibodies in patient plasma. The assay detects antibodies against the receptor-binding protein of the S1 subunit of the SARS-CoV-2 spike protein. A value ≥ 40 arbitrary units per milliliter (AU/ml) was considered evidence of a vaccination response. The Abbott IgG-S Ab results were converted to WHO international units on the basis of circles provided by the manufacturer. The conversion to binding antibody units (BAU) for Abbott was BAU/ml = 0.142 Abbott value in AU/ml.

### Multidimensional flow cytometry

Three milliliters of peripheral blood were collected in EDTA tubes. The samples were subjected to lysis, washed and stained according to the EuroFlow standard sample preparation protocol, adjusted to 10^6^ nucleated cells, and the following tubes were used for standardized staining (as detailed in Supplementary Table 2):

- Tube 1, for the evaluation of the myeloid compartment: CD15, CD14, CD64, CD16, PDL-1, CD33, CD38, HLA-DR, CD45
- Tube 2, for the evaluation of T-cell compartment: CD45RA, CCR7, CD28, CD279, CD27, CD4, CD8, CD3, CD57, CD45.

In each tube, 50 μL of peripheral blood was transferred and incubated for 15 min at room temperature in the dark according to the manufacturer’s instructions.

Unstained controls were used to set the flow cytometer photomultiplier tube voltages, and single-color positive controls were used to adjust the instrument compensation settings. Precision Count BeadsTM were used to obtain absolute counts of cells acquired by flow cytometry. Data from the stained samples were acquired via a Beckman Coulter Navios EX-10 flow cytometer and analyzed via KaluzaTM Software 2.1 (https://www.beckman.it/flowcytometry/software/kaluza-c).

### Computational flow cytometry

FCS files collected from patients diagnosed with lymphoproliferative disorders, both pre- and postvaccination, were analyzed via the cutting-edge algorithm known as "FlowCT" v.1.0.0. This advanced tool represents a semiautomated workflow designed specifically for the deconvolution of immunophenotypic data, enabling objective reporting on extensive data sets. Leveraging automated cell clustering, the algorithm facilitates comprehensive analysis across multiple files, ensuring thorough exploration and interpretation of the collected data [15–17].

Briefly, FCS files were merged, subjected to quality control, normalized through batch removal steps and clustered via FlowSOM (version 2.2.0). Following computational clustering, Infinicyt software (version 2.0; Cytognos SL, Salamanca, Spain, https://www.cytognos.com) was employed for cluster identification. The statistical analysis was subsequently conducted via the output generated by R software (version 4.3.3; https://www.r-project.org/).

### Fecal sample collection

Fecal samples were collected from patients before SARS-CoV-2 vaccination (baseline condition, prevaccination) and (15 days) after vaccination (postvaccination), and 2 samples were obtained from each subject for a total of 30 fecal samples. Fecal samples were collected in sterile tubes, stored in a sealed refrigerated container and delivered to the Medical Molecular Microbiology and Antibiotic Resistance Laboratory (MMARLab), Department of Biomedical and Biotechnological Sciences BIOMETEC of the University of Catania, within 2 h for DNA extraction and sequencing.

### DNA extraction, pcr amplification and sequencing

DNA from the stool samples was extracted with the QIAampPowerFecal Pro DNA Kit (Qiagen, Germany) according to the manufacturer’s protocol, which included mechanical cell lysis with bead beating technology. The quality and quantity of the extracted DNA were checked via a NanoDrop 2000 Spectrophotometer (Thermo Scientific, USA), and the final concentration was confirmed via a Qubit 2.0 fluorometer (dsDNA HS assay, Invitrogen). The bacterial DNA was amplified via the V3‒V4 region of the 16S rRNA gene, the PCR products were purified, and paired-end (2 × 300) sequencing was performed via Illumina MiSeq as previously described [18].

### Microbiome analysis

The QIIME2 (v. qiime2-2021.11) [19] pipeline was used to process the generated raw FASTQ files. First, the paired-end sequences were imported and demultiplexed, and then, quality filtering was performed with the deblurring denoising-16S method [20], which was cut to a quality score of 30. Afterwards, a feature table was constructed, and alpha and beta diversity were obtained by calculating the Shannon entropy, Chao1 index and Pielou evenness and performing Jaccard PCoA. Taxonomy was assigned to the OTUs via the Greengenes Database (version 13_8) at 99% identity. Phyla, genera and species tables were also built, collapsing the feature table and the taxonomy. Differential abundance analyses were performed via two R (v. 4.1.2) packages, namely, MetagenomeSeq (v 1.36) [21] and Limma (v. 3.50.3) [22]. Genera and species were considered statistically significant if the *p* value was < 0.05. For differential abundance analysis, two conditions were analyzed: i) CLL patients before and after vaccination and ii) FL patients before and after vaccination.

### Prediction of metagenome functional content

PICRUSt2 was used for the prediction of metagenomic functions on the basis of the marker gene sequencing profiles of i) CLL patients before and after vaccination and ii) FL patients before and after vaccination [23].

### Statistical analysis

Statistical analyses were performed via GraphPad Prism version 8.0 (GraphPad Software, Inc., San Diego, CA, USA). The data were expressed as the means ± standard deviations or medians with interquartile ranges (IQRs) for the quantitative variables and as numbers and percentages for the categorical variables, where appropriate. Categorical variables were expressed as counts and percentages (%). Groups were compared via the chi-square test or Fisher’s exact test for categorical variables and via Student’s t test or the Mann–Whitney U test for continuous variables. The strength and direction of the associations between immunological parameters were investigated by applying the Pearson correlation coefficient. A two-sided *p* value of less than 0.05 was considered statistically significant.

## Results

### Humoral response to vaccination

Before vaccination, the anti-N antibody was dosed to exclude subjects whose immune system could already be stimulated by previous infections or an ongoing infection (baseline). All the subjects were examined for IgG anti-S titers 15 days after the first (T1) or second dose of vaccine (T2), and only 15 patients were evaluated after the third dose of vaccine (T3).

**Fig. 1:**
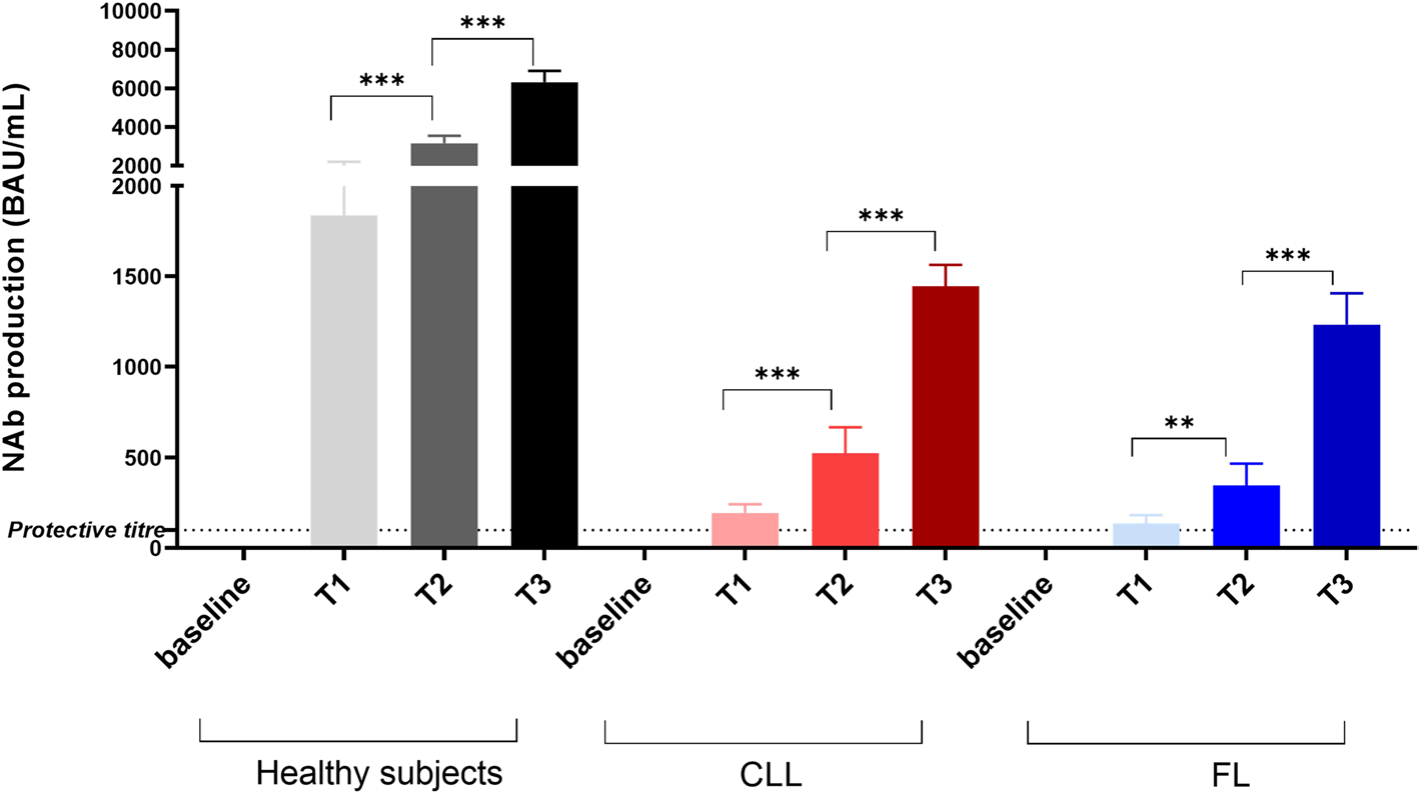
SARS-CoV-2 anti-spike humoral response to COVID-19 vaccination in healthy, CLL and FL subjects. Spike IgG antibodies in healthy (shown in gray gradient), CLL (shown in red gradients) and FL (shown in blue gradient) subjects were evaluated at three different timepoints: before (baseline), 15 days after Dose 1 (T1), 30 days after Dose 2 (T2) and 30 days after Dose 3 (T3) of the BNT162b2 (Pfizer-BioNTech) SARS-CoV-2 vaccine. Antibody concentrations measured in artificial units per mL (AU/mL) were depicted on a log-10 scale to better capture the full range of responses. The dotted reference line represents the threshold at 100 AU/mL corresponding to evidence of a vaccine response. For each subject category (healthy, CLL and FL), a comparison between before and after each dose is shown. Asterisks denote ANOVA significance among t tests for paired samples at each timepoint tested: **p < 0.001, *** *p* < 0.0001. Abbreviations: CLL, chronic lymphatic leukemia; FL, follicular lymphoma; IgG, immunoglobulin IgG class; Nab, neutralizing antibody test; BAU, binding antibody unit

After the first dose of the BNT162b2 vaccine, we confirmed a lower production of antibodies against SARS-CoV-2 in patients with both CLL and FL than in healthy controls (194 ± 48 vs 137 ± 44 vs 1840 ± 361 BAU/mL, respectively; *p* < 0.001, Fig. 1). Ninety percent (18/20) of the non-responder patients had received anti-CD20 treatment in the last 12 months, and none of the patients still receiving treatment with rituximab developed a protective titer.

After the second dose, the production of antibodies against SARS-CoV-2 was still lower in CLL/FL patients than in healthy controls (524 ± 144 vs 347 ± 120 vs 3161 ± 381 BAU/mL, respectively; *p* < 0.001). A total of 14/43 (32%) patients were defined as serological responders since they developed a positive response following the two doses; they received another Nab titer after the third dose. The antibody titer against SARS-CoV-2 was still lower in CLL/FL patients than in healthy controls (1445 ± 119 vs. 1234 ± 171 vs. 6317 ± 581 BAU/mL, *p* < 0.001). Only 4/25 (16%) patients who did not respond to the first dose of vaccine achieved a significant response after the second and third doses.

### Immune profiling in CLL and FL patients after exposure to the BNT162b2 vaccine

**Fig. 2:**
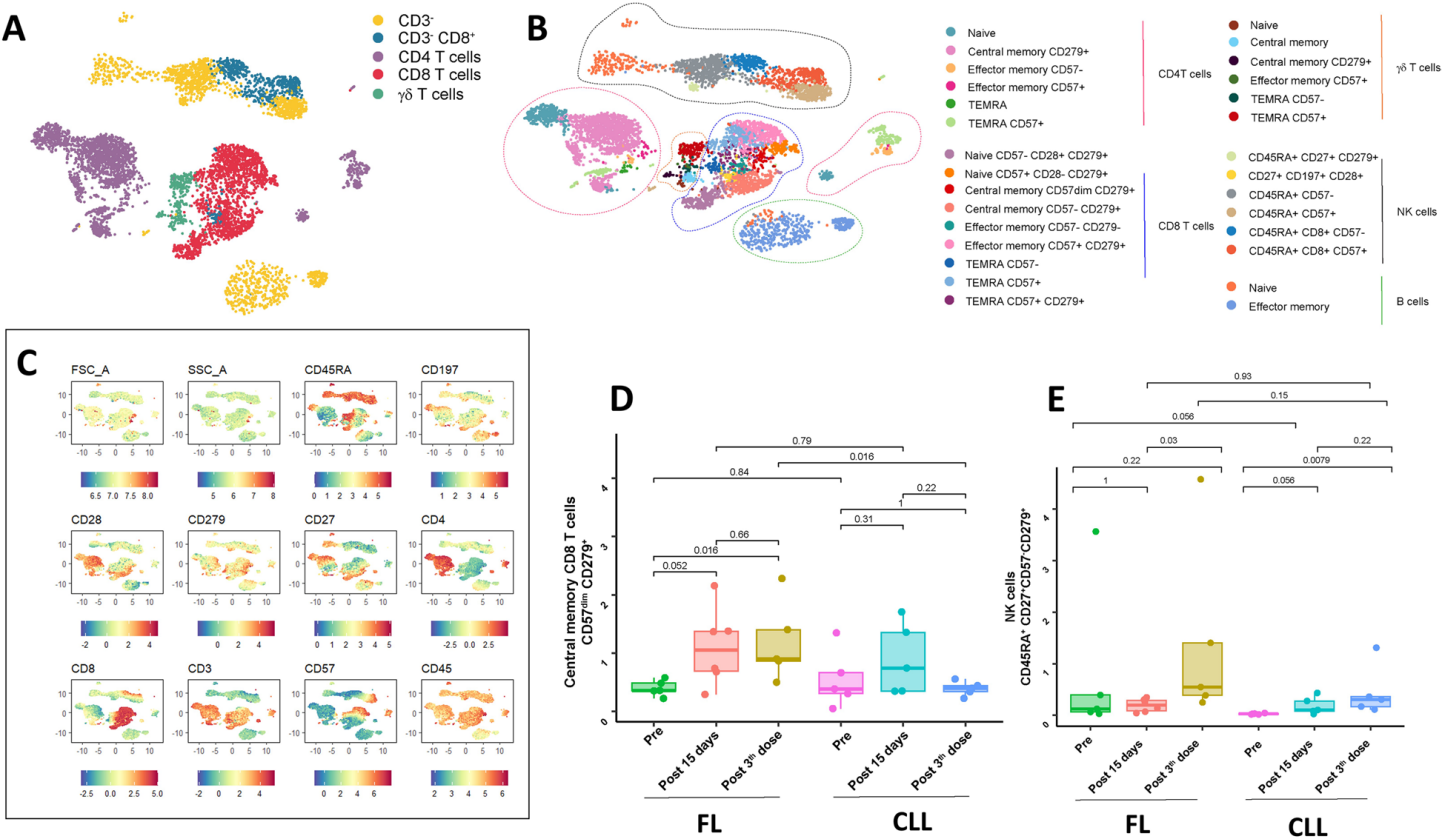
T-cell skewing after COVID-19 vaccination in CLL and FL patients. **A** Uniform manifold approximation and projection (UMAP) of CD3-, CD3- CD8 + , CD4, and CD8 + T cells identified via a self-organizing map (SOM). **B** Characterization of each lymphocyte subset on the basis of the median expression of individual markers in each cell cluster. **C** Uniform manifold approximation and projection (UMAP) illustrating the expression of each marker within immune cell populations identified via a self-organizing map (SOM). **D** Boxplots showing the proportions of central memory CD57^dim^ CD28^+^ CD279^+^ CD8 + T cells and CD45RA^+^ CD27^+^ CD57^−^ CD279^+^ NK cells in FL and CLL patients at baseline, 15 days after Dose 1 (T1), and 30 days after Dose 3 (T3). Abbreviations: CLL, chronic lymphatic leukemia

In the whole cohort of 43 patients, no significant changes in granulocytes or T-cell number or function were detected after 2 vaccine doses (Supplementary Fig. 1). Independent of the vaccine response and therapeutic regimen received, in FL patients, the percentage and absolute number of central memory CD8^+^ CD57^dim^ CD279^+^ T cells were weakly increased 15 days after the first dose (*p* = 0.05) and remained high after the second dose, which was significantly greater than the corresponding measure in CLL patients (*p* = 0.016) (Fig. 2D). In contrast, in the CLL cohort, no significant changes occurred at any tested timepoint.

In both the CLL and FL cohorts, the percentage and absolute number of NK cells with an immunophenotype positive for the expression of CD45RA, CD27 and CD279 and negative for CD 57 increased after 2 doses of vaccination (*p* = 0.03 and *p* = 0.0079, respectively; Fig. 2E).

Based on CD16 and CD14 expression, we followed sequential stages of maturation and distinct functions of monocytes. We detected a weak increase in the number of CD14^+^CD16^+^ monocytes after two doses of vaccination, which was not confirmed after three doses; this increase was associated with reduced expression of the activation marker CD38 but not that of CD64, whose expression was not affected by vaccination (Supplementary Fig. 2).

We identified nine phenotypes in neutrophils sub-populations (Supplementary Fig. 3), significantly increased after 15 days from vaccination in CLL, namely:

- Myeloid-2: CD14^−^CD15^+^CD16^−^CD64^−^CD33^−^CD38^+^PDL1^+^HLA-DR^−^ (*p* = 0.038)
- Myeloid-6: CD14^−^CD15^+^CD16^+^CD64^−^CD33^+^CD38^+^PDL1^+^HLA-DR^−^(*p* = 0.01)
- Myeloid-9: CD14^−^CD15^+^CD16^+^CD64^+^CD33^+^CD38^+^PDL1^+^HLA-DR^+^(*p* = 0.01)

while the Myeloid-1: CD14^−^CD15^+^CD16^dim^CD64^+^CD33^−^CD38^+^PDL1^+^HLA-DR^−^subpopulation was significantly reduced (*p* = 0.007) after 15 days from vaccination in CLL patients.

In FL patients the Myeloid-1 CD14^−^CD15^+^CD16^dim^CD64^+^CD33^−^CD38^+^PDL1^+^HLA-DR^−^subpopulation was reduced after 15 days from vaccination (*p* = 0.01), and increased after one month from vaccination (*p* = 0.013).

### Differences in microbiome composition in CLL pre- and postvaccination

A total of 14 CLL fecal samples from 7 patients before and after SARS-CoV-2 vaccination were sequenced via the Illumina MiSeq platform to determine and compare the microbial profiles before and after vaccination. A total of 1,029,072 valid reads, with an average of 73,505.142 reads/sample, were generated and clustered into 764 OTUs, with at least 97% similarity level using the Greengenes Database.

**Fig. 3:**
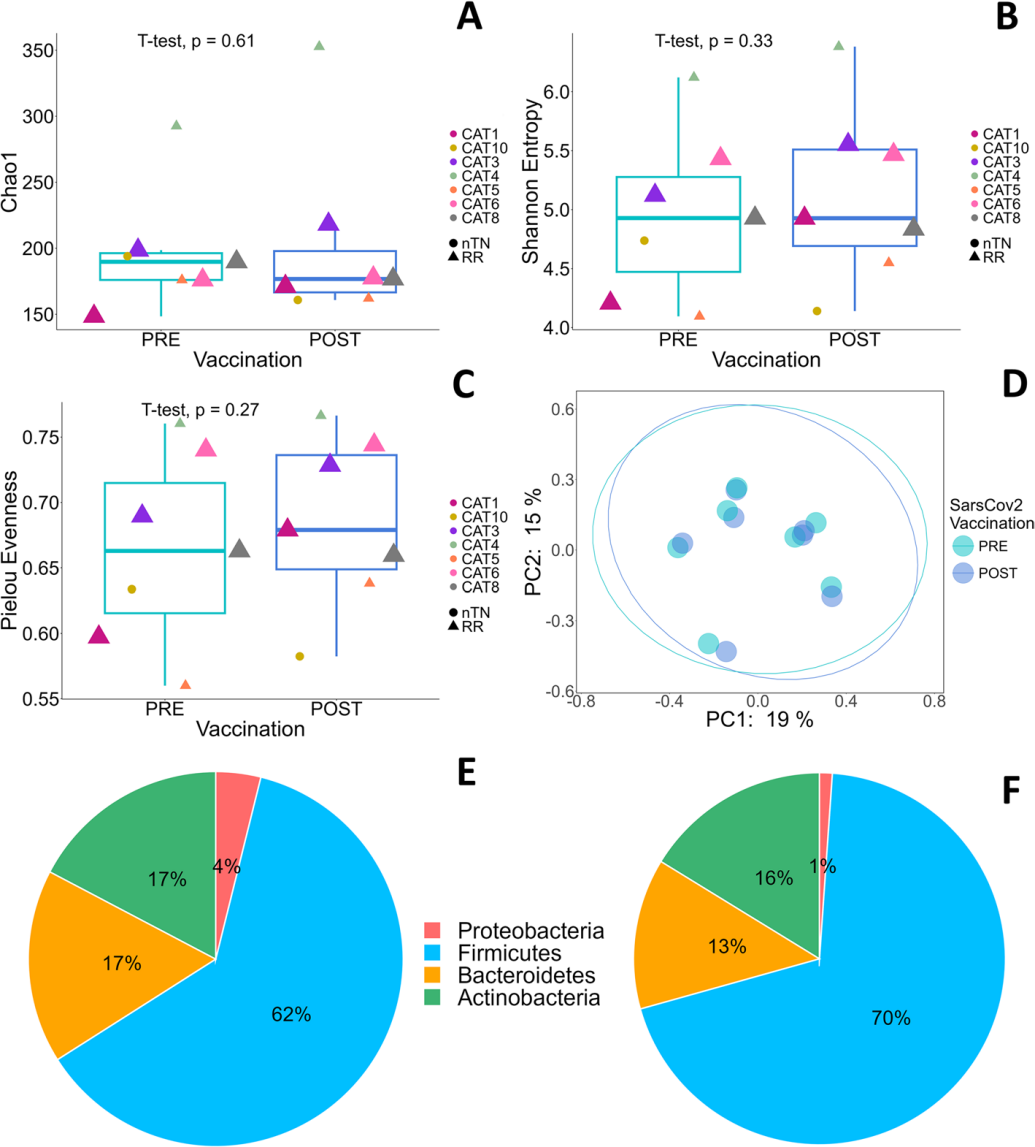
Differences in microbiome composition in patients with chronic lymphatic leukemia before and after COVID-19 vaccination. Comparisons of the microbiome compositions in 7 CLL patients before and after COVID-19 vaccination. **A** Boxplots showing Chao1 index before (green bar) and after vaccination (blue bar). **B** Boxplots showing Shannon entropy index before (green bar) and after vaccination (blue bar). **C** Boxplots showing Pielou evenness index before (green bar) and after vaccination (blue bar). In (**A**), (**B**) and (**C**) plots each point identifies an individual patient, with a unique color code, as shown in the legend. Size refers to the vaccination response 30 days after Dose 3 (small shape size: no response; large shape size: response). Filled circles identify newly diagnosed patients never treated for CLL before baseline evaluation (nTN), whereas triangles identify CLL-relapsed/refractory patients (RR). Additional details are reported in Supplementary Table 2. **D** Jaccard PCoA results for beta diversity: the score plot of PC1 and PC2 does not show a clear separation before (green dots) and after COVID- 19 vaccination (violet dots) in CLL patients 30 days after Dose 3. Relative abundance of phyla in (**E**) CLL patients before vaccination (**E**) and after vaccination (**F**). Abbreviations: CLL, chronic lymphatic leukemia: PC, principal coordinate; nTN: naïve/not treated patients; RR, relapsed/refractory patients

Pre- and postvaccination CLL samples were analyzed in terms of alpha diversity via the Chao1 index (richness), Shannon entropy index (diversity) and Pielou evenness index (diversity among species richness within a community), which revealed no significant differences between pre- and postvaccination fecal samples (*p* > 0.05), although the Chao1 index slightly decreased in the postvaccination group (Fig. 3A, B, C). Instead, the Pielou evenness index increased in the postvaccination group compared with the prevaccination group, suggesting an improvement in microbial diversity after vaccination. Similarly, the overall dissimilarities of the microbial community structure between the two groups were calculated via a principal coordinate analysis (PCoA) plot on a weighted basis (accounting for the abundance of OTUs), which revealed no clustering differentiation of the bacterial communities in the two groups (Fig. 3D). Furthermore, the correlation analysis between alpha diversity and total IgG (Supplementary Fig. 4) was not statistically significant.

The composition of the gut microbiome before and after vaccination revealed four major phyla (Fig. 3E, F). Firmicutes was the most abundant phylum whose abundance further increased after vaccination (from 62 to 70%), Bacteroidetes shifted from 17 to 13%, Actinobacteria from 17 to 16%, and Proteobacteria from 4 to 1%. In any case, no statistically significant differences were found between pre and post-vaccination. Similarly, 93 genera were identified with no statistically significant difference between the two timepoints (Supplementary Fig. 5, Supplementary Table 2), and 120 species were detected with no significant difference between pre- and postvaccination CLL patients.

At the genus level, Collinsella (2.53 vs. 2.86), mainly C. aerofaciens, Blautia (10.17% vs. 11.44%), and Gemmiger (4.38% vs. 6.4%), which belong to the species G. formicilis, Lachnospiraceae (14.05% vs. 22.92%), Ruminococcus (5.2% vs. 6.0%), Streptococcus (1.96% vs. 2.41%), and Prevotella (0.96% vs. 1.22%), mainly P. copri, increased in the postvaccination group compared with the prevaccination group. In contrast, Bacteroides (11.2% vs. 7.55%), mainly B. uniforms, Bifidobacterium (12.84% vs. 11.37%), Oscillatoria (2.77% vs. 1.65%), Faecalibacterium (5.48% vs. 4.33%), *Enterobacteriaceae* (3.16% vs. 0.37%), Lactococcus (2.37% vs. 0.03%), Enterococcus (1.17% vs. 0.18%), and Clostridium (0.28% vs. 0.02%) decreased after vaccination (Supplementary Fig. 3, Table 2). Statistical analysis shown revealed a positive correlation between specific neutrophils subsets and genera affected by vaccination (Supplementary Fig. 6):

- Prevotella and myeloid 2 only post-vaccination (*r* = 0.79; *p* = 0.035),
- Gemmiger and myeloid 1 (*r* = 0.82; *p* = 0.034) only post vaccination
- Bacteroides and myeloid 9 (*r* = 0.8; *p* = 0.034) only prevaccination
- Oscillospira and myeloid 6 in both pre vaccination (*r* = 0.87; *p* = 0.012) and post vaccination (*r* = 0.76; *p* = 0.045) samples.

**Table 2:** Average relative abundances of the top 30 microbial groups (genera and species) before and after COVID-19 vaccination in CLL patients

**Level**	**Group**	**Relative abundance**	
		**Before (%)**	**After (%)**
**Genus**	**↑*Collinsella***	**2.53**	**2.86**
	*Collinsella aerofaciens*	2.26	2.08
	**↑*Blautia***	**10.17**	**11.44**
	**↑*Gemmiger***	**4.38**	**6.4**
	*Gemmigerformicilis*	4.38	6.4
	**↓*Bacteroides***	**11.2**	**7.55**
	*Bacteroidesuniforms*	4.62	2.47
	*Bacteroidesovatus*	0.55	0.39
	*Bacteroidescaccae*	0.29	0.35
	*Bacteroidesfragilis*	0.04	0.19
	**↓ *Oscillospira***	**2.77**	**1.65**
	**↑*Lachnospiraceae***	**14.05**	**22.92**
	**↓*Faecalibacterium***	**5.48**	**4.33**
	*Faecalibacterium prasnitzii*	5.48	4.33
	**↑ *Ruminococcus***	**5.2**	**6.0**
	*Ruminococcus bromii*	3.99	3.82
	*Ruminococcus callidus*	0.14	0.09
	**↑*Dialister***	**0.74**	**1.37**
	**↓ *Bifidobacterium***	**12.84**	**11.37**
	*Bifidobacterium adolescentis*	7.65	2.93
	*Bifidobacterium longum*	3.31	4.67
	**↓*Christensenellaceae***	**0.78**	**0.55**
	**↑*Coprococcus***	**0.04**	**0.14**
	**↑*Streptococcus***	**1.96**	**2.41**
	**↑*Parabacteroides***	**1.16**	**1.25**
	**↑*Suttarella***	**0.10**	**0.17**
	**↓*Bilophila***	**0.23**	**0.18**
	**↓*Enterobacteriaceae***	**3.16**	**0.37**
	*Klebsiella*	0.1	0.0
	**↑*Butyricicoccus***	**0.38**	**0.56**
	*Butyricicoccus pullicaecorum*	0.38	0.56
	**↑*Prevotella***	0.96	1.22
	*Prevotella copri*	0.79	1.06
	**↑***Roseburia*	0.64	0.78
	**↓***Dorea*	0.21	0.14
	**↑*Eggerthella***	**0.27**	**0.3**
	*Eggerthella lenta*	0.27	0.14
	**↓*Lactococcus***	**2.37**	**0.03**
	**↓*Enterococcus***	**1.17**	**0.18**
	**↑*Lactobacillus***	**0.07**	**0.7**
	*Lactobacillus delbrueckii*	0.0	0.06
	*Lactobacillus mucosae*	0.0	0.26
	**↓*Actinomyces***	**0.11**	**0.08**
	**↓* Clostridium***	**0.28**	**0.02**
	*Clostridium perfringens*	0.05	0.14
	**↓ *Haemophilus***	**0.1**	**0.04**
	*Haemophilus parainfluenzae*	0.1	0.04
	**↓*Butyrivibrio***	**0.19**	**0.03**
	**↓*Alistipes***	**0.65**	**0.27**
	*Alistipesindistinctus*	0.02	0.02
	*Alistipesfinegoldii*	0.08	0.07
	*Alistipesmassiliensis*	0.13	0.06

The arrows indicate the direction of the variation with respect to the baseline evaluation: ↓, decreased; ↑, increased; = , unchanged

### Differences in the microbiome composition of FL pre- and postvaccination

**Fig. 4:**
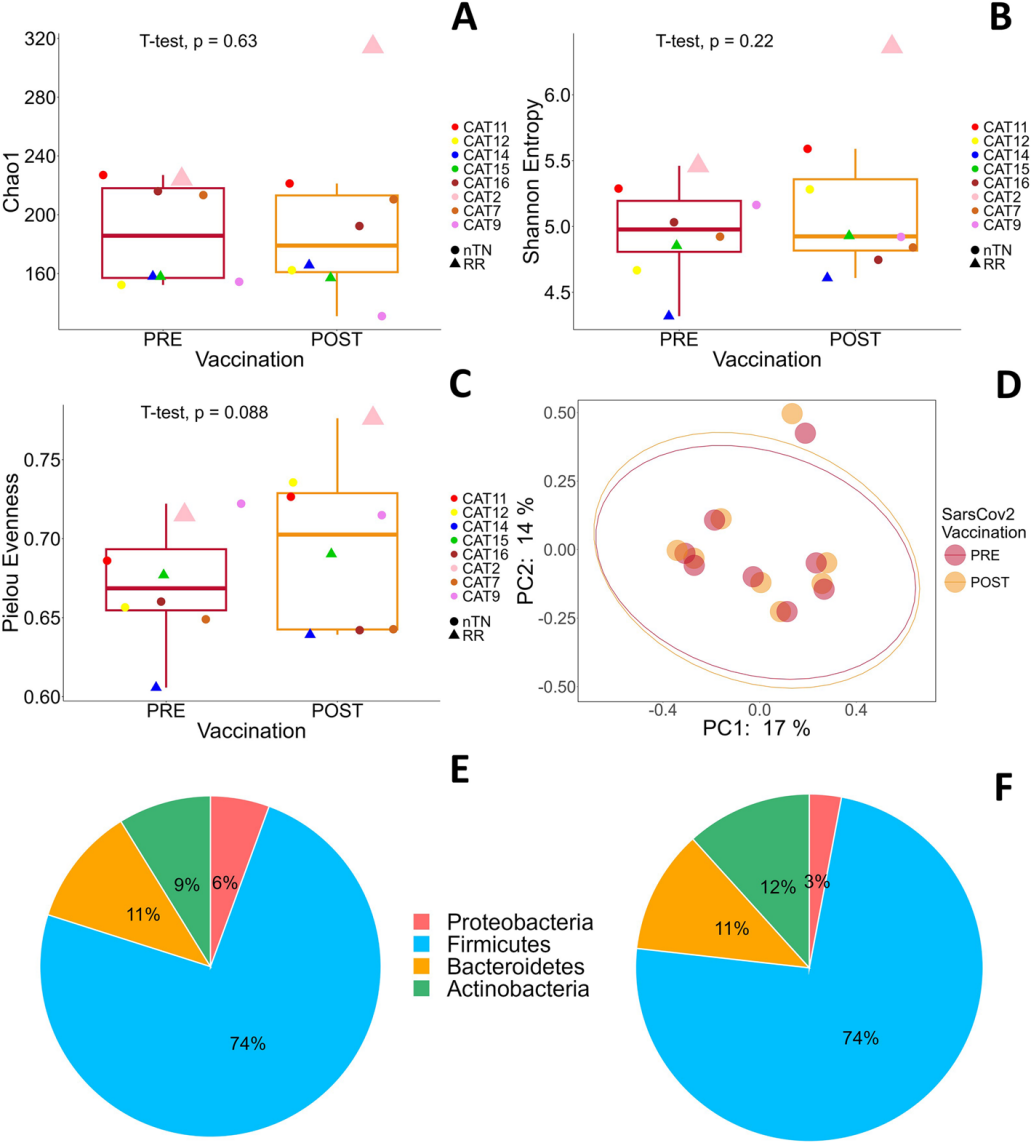
Differences in microbiome composition in patients affected by follicular lymphoma before and after COVID-19 vaccination. Comparisons of microbiome composition in 8 FL patients before and after COVID-19 vaccination. **A** Boxplots showing Chao1 index before (red bar) and after vaccination (orange bar). **B** Boxplots showing Shannon entropy index before (red bar) and after vaccination (orange bar). **C** Boxplots showing Pielou evenness index before (red bar) and after vaccination (orange bar). In (**A**), (**B**) and (**C**) plots each point identifies an individual patient, with a unique color code, as shown in the legend. Size refers to the vaccination response 30 days after Dose 3 (small shape size: no response; large shape size: response, achieved by only 1 RR patient). Filled circles identify newly diagnosed patients never treated for FL before baseline evaluation (5, nTN), whereas triangles identify FL- relapsed/refractory patients (3, RR). Additional details are reported in Supplementary Table 2. **D** Jaccard PCoA results for beta diversity: the score plot of PC1 and PC2 does not show a clear separation before (green dots) and after COVID-19 vaccination (violet dots) in FL patients 30 days after Dose 3. Relative abundances of phyla in FL patients before vaccination (**E**) and after vaccination (**F**). Abbreviations: FL, follicular lymphoma; PC, principal component; nTN: naïve/not treated patients; RR, relapsed/refractory patients

16 FL fecal samples from 8 patients before and after SARS-CoV-2 vaccination were sequenced via the Illumina MiSeq platform. A total of 1,137,010 valid reads with an average of 71,063.125 reads/sample were generated and clustered into 720 OTUs, with at least 97% similarity level using the Greengenes Database. Like for CLL samples, the alpha diversity did not differ in terms of the richness of the microbial community according to the Chao1 and Shannon entropy indices in the FL pre- and postvaccination groups (Fig. 4 A-B), but only the Pielou evenness index (Fig. 4C) was appreciably greater in the postvaccination group than in the prevaccination group, strengthening the hypothesis. The principal coordinate analysis (PCoA) for beta diversity analysis revealed that both conditions clustered together (Fig. 4D), suggesting that FL before and after vaccination were very similar.

The microbial profiles revealed four major phyla, with no statistically significant differences at the two different timepoints (Fig. 4E-F). Firmicutes was the most abundant group, accounting for 74% of the bacteria before and after vaccination, and Bacteroidetes remained the same under the two conditions (11%). Conversely, Actinobacteria decreased from 12 to 9%, and Proteobacteria was halved from 6 to 3%. At the genus level, we identified 77 genera (Supplementary Fig. 7), but there was no statistically significant difference between pre- and postvaccination FL patients; likewise, we detected 115 species with no statistically significant changes. Some genera increased after vaccination compared to prevaccination, namely the genera Collinsella (5.22% vs. 6.13%), mainly C. aerofaciens, Bacteroides (6.57% vs. 7.2%), Gemmiger (6.56% vs. 7.31%), Lachnospiraceae (18.43% vs. 20.13%), Bifidobacterium (1.99% vs. 3.7%), Parabacteroides (0.58% vs. 0.75%), Sutterella (0.09% vs. 0.13%), Bilophila (0.09% vs. 0.15), and Dorea (0.14% vs. 0.35%). Conversely, Dialister (1.23% vs. 0.72%), Streptococcus (7.67% vs. 4.33%), Roseburia (1.68% vs. 0.93%), Enterobacteriaceae (5.29% vs. 2.5%), Prevotella (2.47% vs. 0.9%), mainly P. copri, Lactobacillus (0.35% vs. 2.44%), Enterococcus (0.16% vs. 0.02%), Coprococcus (1.2% vs. 0.44%) and Weissella (0.13 vs. 0%) decreased in postvaccination patients (Supplementary Fig. 7, Table 3).

**Table 3:** Average relative abundances of the top 30 microbial groups (genera and species) before and after COVID-19 vaccination in FL patients

**Level**	**Group**	**Relative abundance**	
		**Before (%)**	**After (%)**
**Genus**	**↑*Collinsella***	**5.22**	**6.13**
	*Collinsella aerofaciens*	5.22	6.13
	**↑*Blautia***	**10.11**	**10.7**
	**↑*Gemmiger***	**6.56**	**7.31**
	*Gemmigerformicilis*	6.56	7.31
	**↓*Bacteroides***	**6.57**	**7.2**
	*Bacteroidesuniforms*	1.93	1.94
	*Bacteroidesovatus*	0.23	0.28
	*Bacteroidescaccae*	0.42	0.36
	*Bacteroides fragilis*	0.1	0.11
	**↑*Sutterella***	**0.09**	**0.13**
	**↑*Lachnospiraceae***	**18.43**	**20.13**
	**↓*Faecalibacterium***	**12.43**	**11.72**
	*Faecalibacterium prasnitzii*	12.4	11.72
	**↑ *Ruminococcus***	**4.52**	**4.82**
	*Ruminococcus bromii*	3.58	3.96
	*Ruminococcus callidus*	0.44	0.17
	**↓*Dialister***	**1.23**	**0.72**
	**↑Bifidobacterium**	**1.99**	**3.7**
	*Bifidobacterium adolescentis*	0.19	1.14
	*Bifidobacterium longum*	1.38	1.02
	**↑*Christensenellaceae***	**0.44**	**0.62**
	**↓*Coprococcus***	**1.2**	**0.44**
	**↓*Streptococcus***	**7.67**	**4.33**
	**↑*Parabacteroides***	**0.58**	**0.75**
	*Parabacteroides distasonis*	**0.22**	**0.32**
	**↑*Alistipes***	**0.06**	**0.16**
	**↑*Bilophila***	**0.09**	**0.15**
	**↓*Enterobacteriaceae***	**5.29**	**2.5**
	**↓*Butyricicoccus***	**0.59**	**0.55**
	*Butyricicoccus pullicaecorum*	0.59	0.55
	**↓*Prevotella***	2.47	0.9
	*Prevotella copri*	2.31	0.65
	**↓*Roseburia***	**1.68**	**0.93**
	**↑*Dorea***	**0.14**	**0.35**
	*Dorea formicigenerans*	0.14	0.35
	**↑*Eggerthella***	**0.1**	**0.13**
	*Eggerthella lenta*	**0.1**	**0.13**
	**↑*Lactococcus***	**0.02**	**0.04**
	**↓*Enterococcus***	**0.16**	**0.02**
	**↑*Lactobacillus***	**0.35**	**2.44**
	*Lactobacillus delbrueckii*	0.12	0.0
	*Lactobacillus mucosae*	0.05	0.25
	= ***Actinomyces***	**0.05**	**0.05**
	**↓*Weissella***	**0.13**	**0.0**
	**↑*Megamonas***	**0.02**	**0.11**
	**↑*Paraprevotella***	**0.11**	**0.17**
	**↓*Slackia***	**0.04**	**0.01**

The arrows indicate the direction of the variation with respect to the baseline evaluation: ↓, decreased; ↑, increased; = , unchanged

The correlation analysis between alpha diversity of FL and total IgG (Supplementary Fig. 8) was not statistically significant. Differently from CLL patients, the statistical analysis between alpha diversity and immunological subsets revealed a negative correlation between Shannon entropy and Pielou evenness indices and frequence of myeloid 1 (respectively, *r* = −0.73; *p* = 0.039 and *r* = −0.74; *p* = 0.037) and myeloid 5 (respectively*, r* = −0.77; *p* = 0.026 and *r* = −0.74; *p* = 0.035) subsets in pre vaccination; while in post vaccination the Shannon entropy was negatively correlated only with myeloid 6 (*r* = −0.79; *p* = 0.02) and Chao1 with myeloid 6 (*r* = −0.81; *p* = 0.016) subsets (Supplementary Fig. 9).

In addition, in post vaccination samples we found a negative correlation between Bifidobacterium and myeloid 4 subsets (*r* = −0.76; *p* = 0.037) and a positive correlation between Lactobacillus abundance and myeloid 1 frequence subsets (*r* = 0.72; *p* = 0.044). Despite myeloid 2 subset did not change in FL patients after vaccination, we found a positive correlation in both samples analysed before and after vaccination with Weissella abundance (Supplementary Fig. 10).

### Metabolic differences by PICRUSt2 analysis

**Fig. 5:**
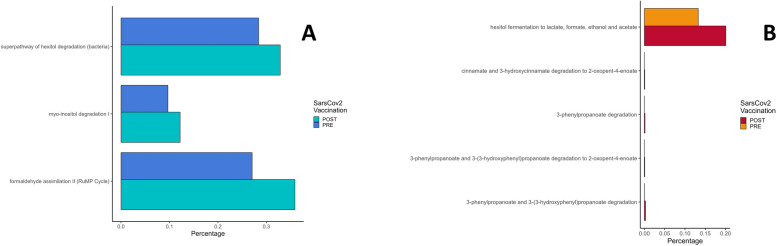
Metabolic differences driven by microbiome communities in the CLL and FL cohorts. Metabolic differences before and after COVID-19 vaccination were calculated via PICRUSt2 with MetaCyc pathways. **A** Bars showing percentage of metabolic differences before (dark blue) and after vaccination (light blue) in CLL patients. **B** Bars showing percentage of metabolic differences before (dark red) and after vaccination (orange) in FL patients. Abbreviations: CLL, chronic lymphatic leukemia; FL, follicular lymphoma

The PICRUSt2 analysis used to predict the functional potential of microbial communities revealed three pathways with statistically significant differences in the pre- and postvaccination CLL groups: the superpathway of hexitol degradation (logFC 0.69) and myo-inositol degradation (logFC 0.88), both involved in the catabolism of sugars, and the formaldehyde assimilation or RuMP cycle (logFC 0.73), which can lead to either fixation or detoxification of formaldehyde (Fig. 5A).

Under pre- and postvaccination FL conditions, several statistically significant pathways were involved in the catabolism of phenylpropanoid compounds and were more represented in the postvaccination cohort: 3-phenylpropanoate degradation (logFC 3.74), 3-phenylpropanoate and 3-(3-hydroxyphenyl) propanoate degradation (logFC 3.82), 3-phenylpropanoate and 3-(3-hydroxyphenyl)propanoate degradation to 2-oxopent-4-enoate (logFC 3.17) and cinnamate and 3-hydroxycinnamate degradation to 2-oxopent-4-enoate (logFC 3.17), which all converge to the tricarboxylic acid cycle. Heterolactic fermentation was statistically overrepresented under postvaccination conditions, expressed as hexitol fermentation to lactate, formate, ethanol and acetate pathways, with a logFC of 0.79 (Fig. 5B).

### Comparison of the CLL and FL groups pre- and postvaccination

**Fig. 6:**
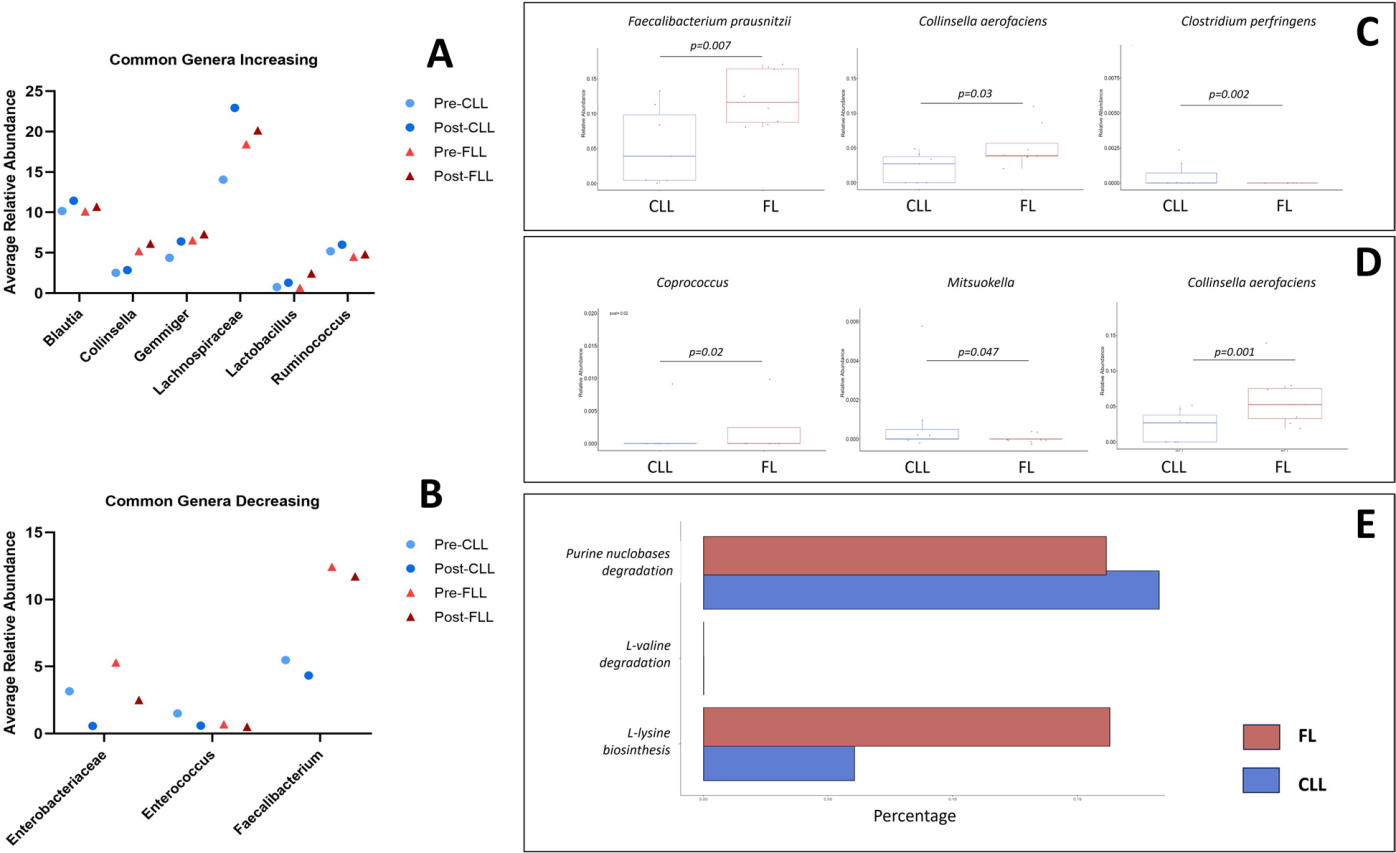
Changes in microbiome communities and metabolic pathways in the CLL and FL cohorts before and after COVID-19 vaccination. **A** Bars showing genera abundance percentage before (dark blue) and after vaccination (light blue) in CL patients. **B** Bars showing genera abundance percentage before (dark red) and after vaccination (orange) in FL patients. When CLL and FL were compared before COVID-19 vaccination, statistical differences in the relative abundance of species in CLL and FL patients were observed (**C**). Similarly, 30 days after 3 COVID-19 vaccination statistical differences in the relative abundances of genera and species among CLL and FL patients were observed (**D**). After COVID-19 vaccination, metabolic differences in CLL and FL patients were calculated via PICRUSt2 with MetaCyc pathways (**E**). Abbreviations: CLL, chronic lymphatic leukemia; FL, follicular lymphoma

When the two cohorts were compared after vaccination, the genera Collinsella, Gemmiger, Lachnospiraceae, Blautia, Ruminococcus and Lactobacillus increased in both CLL patients and FL patients (Fig. 6A), whereas Faecalibacterium, Enterobacteriacae, and Enterococcus decreased (Fig. 6B). Only when comparing the two different pathologies we found some statistically significant species that differed between CLL and FL: Collinsella aerofaciens and Faecalibacterium prausnitzii were more abundant in FL prevaccination than in CLL, whereas Clostridium perfringens was less represented (Fig. 6C). In the postvaccination samples, only F. prausnitzii and C. aerofaciens were significantly more abundant in FL than in CLL (Fig. 6D). Multivariate analysis failed to identify factors associated with changes in microbiome communities among the CLL and FL cohorts, considering age, sex, exposure to anti-CD20 therapy and disease activity.

PICRUSt2 analysis allowed the identification of metabolic pathways that were significantly different between CLL and FL patients postvaccination. The L-lysine biosynthesis pathway was more represented in CLL patients (logFC of 1.25), whereas the L-valine degradation pathway (logFC of −2.00) and the anaerobic degradation of purine nucleobases (logFC of −0.63) were overrepresented in the FL cohort (Fig. 6E).

## Discussion

This longitudinal prospective study highlights the integrative changes in immune function and microbiome composition in CLL and FL patients to contribute to future risk assessments, management strategies and the timing of mRNA-based vaccination for this highly immunocompromised cohort of patients.

Our findings are in line with those of previous reports on large cohorts. In the Israelian series of 2030 CLL patients receiving 2 doses of the BNT162b2 vaccine, the protective effect of vaccination was 46% [24], which was lower than the 95% protection reported in the pivotal phase III BNT162b2 vaccine trial [25]. One week after the second BNT162b2 vaccine dose, the risk of SARS-CoV-2 infection and symptomatic COVID-19 decreased by 46% and 40%, respectively, and the number of COVID-19–related deaths declined to 16.7% [24]. The antibody-mediated response to the BNT162b2 mRNA COVID-19 vaccine in patients with CLL was markedly impaired and affected by disease activity and treatment. Indeed, in patients treated with either Bruton’s tyrosine kinase inhibitors or venetoclax ± anti-CD20 antibody, response rates were considerably low (16.0% and 13.6%), with no response in patients exposed to anti-CD20 antibodies < 12 months before vaccination [24, 26, 27]. In a small series of 14 CLL patients treated with ibrutinib and vaccinated with BNT162b2, the T-cell response to spike peptides was more blunted in CLL patients treated with ibrutinib than in untreated patients [28]. Higher levels of IFNγ and TNFα secretion by CD3 + CD4 + T cells were detected in treatment-naïve patients than in ibrutinib-treated patients before vaccination, but no significant difference between these two groups was detected after SARS-CoV-2 vaccination [29]. In patients receiving ibrutinib, humoral immunity was reduced, with markedly low anti-spike IgG and virtually no neutralizing activity to the delta and omicron variants [26–28].

In the FL setting, therapies, rather than the disease process, are detrimental to seroconversion to the BNT162b2 vaccine [29]. Despite their poor humoral response, FL patients had measurable antigen- specific CD4 + and CD8 + T-cell responses of a magnitude comparable to those of healthy controls [29], suggesting the presence of additional immunological circuitries to control the immune response, including a bidirectional relationship between the gut microbiota and the COVID-19 vaccine, with the varying components of the microbiota either enhancing or reducing the vaccine’s efficacy. To our knowledge, this is the first longitudinal, prospective study able to identify a distinct immune and microbiome signature in CLL and FL patients after receiving the BNT162b2 vaccine.

In the CLL cohort, at baseline we detected increased abundances of the families Bacteroidaceae (11.2%), Ruminococcaeae, Lachnospiraceae, as previously reported [30], and Bifidobacteriaceae, with the most abundant species being Bifidobacterium adolescentis, confirming the findings of a previous report that showed elevated relative abundance of intestinal Bacteroides and decreased Firmicutes in CLL patients compared to healthy controls, suggesting a loss of complexity of the microbiome reflecting the antigen-driven distortion of the immune system [30, 31]. After vaccination in CLL patients, independent of seroconversion, the strongest depletion involved Bifidobacterium adolescentis, previously reported as the most abundant in healthy individuals with strong response to the BNT162b2 mRNA COVID-19 vaccine [32].

Oscillospira, member of the family Ruminococcaceae, order Clostridiales, class Clostridia in the phylum Firmicutes was among the most abundant genera, and it positively correlated with myeloid 6 subsets before and after vaccination in the CLL cohort. Within the Ruminococcaeae family, the genus Gemmiger, which was analyzed at the species level, was identified as G. formicilis as the main component in both CLL and FL. This species is characterized by the production of formic acid, butyrate, and lactate [33], essential for anti-inflammatory effects through the inhibition of histone deacetylase in several immune cells [34] and the cross-feeding activity between formate-producing and acetate- producing species [35]. In our study, Gemmiger positively correlated with abundance of neutrophils subset myeloid 1 in postvaccination in CLL patients.

On the opposite, in the FL cohort, Firmicutes was the most abundant phylum, in samples collected before and after vaccination, reaching approximately 70% relative abundance, differing from what was previously described by Diefenbach et al. [36], who reported how microbial dysbiosis was associated to lymphoma patients, with an increase in Bacteroidetes and a decrease in Firmicutes at the phylum level. The Ruminococcaeae family, recently addressed as protective in FL setting [37], was increased after vaccination.

Despite the inherent differences in these two pathologies, when gut microbiome of pre- and post- vaccination was compared in each cohort, similar shifts were observed after two doses of the BNT162b2 vaccine, indicating a trend for certain genera in both CLL and FL. Among these we found an increase of certain genera such as Lactobacillus spp., involved in the production of short-chain fatty acids (SCFAs: acetate, propionate, and butyrate), which are known for enhancing B-cell metabolism, promoting differentiation into antibody-producing cells, with anti-inflammatory effects in mice [38, 39]. Lactobacillus increased approximately tenfold after vaccination in both CLL and FL patients, even if we found that alpha-diversity was significantly associated to neutrophil subsets (myeloid 1 and 5) only in FL patients. The genus Lactobacillus exerts its probiotic activity by reinforcing intestinal barrier function [40], by producing conjugated linolenic acid and extracellular polysaccharides to boost cancerous cells apoptosis [37]. In the Lactobacillaceae family, Weissella was mostly represented in FL and it positively correlated with myeloid 2 in samples collected before and after vaccination. Similarly, Lachnospiraceae is a family that includes many species involved in the conversion of primary to secondary bile acids, the production of different SCFAs (acetate, propionate, and butyrate), the prevention of pathogen colonization through the production of propionate, recently associated with FL lymphomagenesis [37]. Lachnospiraceae abundantly synthesize butyrate via several intricate metabolic routes, interfering with gastrointestinal lymphoma reinforcing the NF-κB pathway via the MyD88-dependent TLR4 signaling [41].

The genus Collinsella increased after vaccination in both CLL and FL patients, but the main species Collinsella aerofaciens was statistically more represented in FL in pre and postvaccination. C. aerofaciens is a major producer of ursodeoxycholic acid, a secondary bile acid of interest for its ability to suppress proinflammatory cytokines such as TNF-α, IL-1β, IL-2, IL-4, and IL-6 and cytokine storm syndrome in COVID-19 infections [42], suggesting an association between the increase in this species and a stronger immunological response. Only three groups decreased in CLL and FL patients after vaccination, namely Enterobacteriaceae, Enterococcus and Faecalibacterium. Enterobacteriaceae were depleted in CLL and FL, which could have a positive effect on reducing the inflammatory status at mucosal levels, as previously shown in bowel inflammatory diseases [43, 44]. The genus Enterococcus decreased in both CLL and FL. In addition to its metabolic role as a commensal bacterium, Enterococcus can also impact on the human immune system by playing an immunomodulatory role and activating CD4 + T cells, CD8 + T cells and B lymphocytes, as shown by *E. faecalis*. Nonetheless, in cases of dysbiosis, these bacteria are also implicated in crossing the intestinal barrier, translocating to the bloodstream and causing bacteremia [45]. Faecalibacterium was depleted after vaccination in both groups, and it was identified at the species level by Faecalibacterium prausnitzii [46]. This species is usually associated with health-promoting effects, as it is the main butyrate producer in the gut microbiome, exerting anti- inflammatory effects [47]. On the other hand, F. prausnitzii was significantly more abundant in the FL cohort before vaccination than in the other cohorts and responded better overall to vaccination. Compared with the control, F. prausnitzii might be associated with the production of the secondary bile acid lithocolic acid (LCA) [48], and in another study on H1N1 vaccination, a 1000-fold reduction in LCA was associated with the worst response to vaccination, inflammatory signaling and a disturbed plasma metabolome [35]. The genus Prevotella showed a different behavior in the two cohorts, as it increased in CLL, positively associated with an increase in neutrophil subset myeloid 2, and decreased in FL after vaccination. Prevotella is a pathobiont of the gut microbiota, as it can mediate mucosal inflammation that can lead to systemic inflammation [49].

Being out of scope of this work addressing the function of neutrophil subsets myeloid 1 and 5, we could speculate that the expansion of tumour-associated neutrophils is linked to microbiome composition and is potentially targetable, based on our evidence that BNT162b2 vaccine was able to induce changes in abundance of specific genera correlated with modulation of specific neutrophils subsets. In solid cancers, microbial therapy can promote the conversion of tumor-associated-neutrophils from wound healing to an acutely activated cytotoxic phenotype, an effect poorly investigated in FL and CLL settings [50].

On the other hand, the association between changes occurring in immunome and microbiome composition upon vaccination in CLL and FL patients could reflect variability in diet restriction, antibiotic exposure, age and obesity, factors that we took in account, despite the low numbers of recruited patient in both cohorts, the major limit of our work. Multivariate analysis failed to identify factors associated with changes in microbiome communities among the CLL and FL cohorts, considering age, sex, exposure to anti-CD20 therapy and disease activity.

Functional metabolic analysis by PICRUSt revealed several statistically significant pathways increased in CLL and FL patients postvaccination when compared to the respective pre-vaccination samples. In CLL, these pathways are involved in bacterial metabolism related to sugar degradation and the subsequent production of SCFAs, and formaldehyde assimilation, which is likely involved in cross-feeding between formate-producing and acetate-producing bacteria in the circuitry of SCFA production [51]. Similarly, in FL several pathways are involved in the TCA cycle, where intermediate metabolites can also be used for SCFA production [51]; moreover, we detected an increase in the hexitol fermentation pathway, which is involved in the production of formate, among other metabolites. These differences might be due to vaccination but also the inherent differences between CLL and FL. Presently, the L-lysine biosynthesis was overrepresented in CLL compared to FL, which could have implications in the functionality of the gut microbiome, as lysine acetylation might regulate SCFA production in gut microbiome especially in the Firmicutes phylum [52], and an increase in lysine production could be involved in an increase of SCFA production. Moreover, CLL is characterized by metabolic alterations in the lymph node microenvironment involving amino acid pathways [53], but this could not exclude an ulterior role played by the microbial counterpart in these patients. For the FL cohort two pathways were overrepresented compared to CLL after vaccination. The L-valine degradation pathway is an important catabolic pathway that use branched-chain amino acids (BCAAs) like valine as alternative energy sources in mammals and bacteria, producing energy rich-intermediates like acetyl-CoA and propionyl-CoA [54]. Whereas an increase in serum levels of BCAAs is involved in metabolic diseases [55], an increase in the catabolism of BCAAs might be due to certain feature species in FL compared to CLL, and could be linked to a lower level of circulating BCAAs as they are degraded by gut microorganisms. The second pathway statistically significant in FL was the anaerobic degradation of purine nucleobases, another catabolic pathway involved in the production of metabolic intermediates like uric acid. As gut microorganisms are involved in the host global purine homeostasis and uric acid levels in the serum, the microbiome can also affect the host health like in inflammatory diseases [56]. This feature could also be specific to FL pathology compared to CLL [57, 58].

## Conclusions

Our study confirmed the effect of the BNT162b2 vaccine on shaping the microbiome composition in CLL and FL patients, despite receiving treatment for their underlying active disease, with increases in the genera Collinsella, Gemmiger, Lachnospiraceae, Blautia, Ruminococcus and Lactobacillus and decreases in Faecalibacterium, Enterobacteriaceae, and Enterococcus. Taken together, our data highlights the importance of future comprehensive analysis of the immunome and microbiome profiles to understand immune function in FL and CLL patients.

## Supplementary Information

Supplementary Material 1.

## Data Availability

Data is provided within the manuscript or supplementary information files.
