# Supplementary material for: BNT162b2 mRNA vaccination affects the gut microbiome composition of patients with follicular lymphoma and chronic lymphocytic leukemia
Source: Biomark Res. 2025 Feb 10;13:25. doi: 10.1186/s40364-025-00734-w (PMC11812150; doi:10.1186/s40364-025-00734-w)
Supplement: Supplementary file 1 — Supplementary Material 1. [file 40364_2025_734_MOESM1_ESM.pdf]

BNT162b2 mRNA vaccination affects the gut microbiome composition of patients with follicular lymphoma and chronic lymphocytic leukemia

Annalisa Chiarenza[1*], Gaia Vertillo Aluisio[2*], Nunziatina Laura Parrinello[1], Sara Marino[3], Anna Maria Corsale[4], Grete Francesca Privitera[5], MojtabaShekarkar Azgomi[4], Enrico La Spina[3], Daniela Cambria[3], Angelo Curtopelle[1], Gaetano Isola[3], Cirino Botta[4], Francesco Di Raimondo[1,3] Alessandra Romano[1,3#], Maria Santagati[2#]

Supplementary Materials

List of supplementary materials

Supplementary Table 1: Patients' disposal and response to vaccination
Supplementary Table 2: Details of the monoclonal antibodies used throughout the study
Supplementary Figure 1
Immune subsets in FL and CLL at different timepoints after COVID-19 vaccination
Supplementary Figure 2
Monocyte subsets in FL and CLL at different timepoints after COVID-19 vaccination
Supplementary Figure 3
Neutrophil subsets in FL and CLL at different timepoints after COVID-19 vaccination
Supplementary Figure 4
Evaluation of alpha diversity based on total amount of IgG before and after COVID-19 vaccination in CLL patients
Supplementary Figure 5
Bacterial genera abundance percentages in CLL patients before and after vaccination
Supplementary Figure 6
Correlation between specific neutrophils subsets affected by vaccination and genera abundance in CLL patients
Supplementary Figure 7
Bacterial genera abundance percentages in FL patients before and after vaccination
Supplementary Figure 8
Evaluation of alpha diversity based on total amount of IgG before and after COVID-19 vaccination in FL patients
Supplementary Figure 9
Correlations between alpha-diversity indices and frequence of myeloid subsets in FL patients
Supplementary Figure 10
Correlation between specific neutrophils subsets affected by vaccination and genera abundance in FL patients

Supplementary Table 1: Patients' disposal and response to vaccination

Patient ID	Disease	Status disease	Response to vaccine	Last treatment	Treatment ongoing	Hypogammaglobulinemia	Immunoglobulin replacement
CAT_1	CLL	RR	responder	Venetoclax single agent	Yes, venetoclax single agent	no	no
CAT_3	CLL	RR	responder	R-venetoclax	Yes, R-venetoclax	yes	no
CAT_4	CLL	RR	not responder	R-chemo	No, off therapy	no	no
CAT_5	CLL	RR	not responder	idelalisib + rituximab	Yes, continuous therapy	yes	yes
CAT_6	CLL	RR	responder	Ibrutinib	Yes, continuous therapy	no	no
CAT_8	CLL	RR	responder	R-venetoclax	Yes, continuous therapy	yes	yes
CAT_10	CLL	nTN	not responder	obinutuzumab + chemo	No, off therapy	yes	yes
CAT_2	FL	RR	responder	R-chemo	No, off therapy	yes	yes
CAT_7	FL	nTN	not responder	R-chemo	No, off therapy	yes	yes
CAT_9	FL	nTN	not responder	obinutuzumab + chemo	No, off therapy	no	no
CAT_11	FL	nTN	not responder	R-chemo, R maintenance	Yes, continuous therapy	yes	no
CAT_12	FL	nTN	not responder	obinutuzumab	Yes, continuous therapy	yes	yes
CAT_13	FL	nTN	responder	R-chemo, R maintenance	Yes, continuous therapy	yes	no
CAT_14	FL	RR	not responder	R-chemo (BR)	No, off therapy	no	no
CAT_15	FL	RR	not responder	R-chemo (BR)	Yes, R-chemo	no	no
CAT_16	FL	nTN	not responder	R-chemo	Yes, R-chemo	no	no

Abbreviations: CLL, chronic lymphatic leukemia, FL, follicular lymphoma, nTN, relapse after previous regimen, RR, relapsed/refractory disease, R, Rituximab, BR, bendamustine+rituximab

Supplementary Table 2: Details of the monoclonal antibodies used throughout the study

	Protein	Fluorochrome	Manufacturer	Catalog	Lot number	Clone	Reactivity
Tube 1	CD15	FITC	Beckman	B36298	200027	80h5	Human
	CD14	PE	Beckman	A07764	200059	RMO52	Human
	CD64	ECD	Beckman	A98434	200069	22	Human
	CD16	PC5	Beckman	A07767	200057	3G8	Human
	PD-L1	PC7	Beckman	A78885	200050	PD-L1.3.1	Human
	CD33	APC	Beckman	IM2471	200075	D3HL60.251	Human
	CD38	ALEXA-750	Beckman	B49200	200027	LS198-4-3	Human
	HLA-DR	Pacific Blue	Beckman	B36291	200065	Immu-357	Human
	CD45	Krome Orange	Beckman	A96416	200109	J33	Human
Tube 2	CD45RA	FITC	Beckman	B53328	081124_01	2H4LDH11LDB9	Human
	CCR7	PE	Beckman	B53328	081124_01	G043H7	Human
	CD28	ECD	Beckman	B53328	081124_01		Human
	CD279/PD1	PC5.5	Beckman	T130-117-681	081124_01	PD1.3	Human
	CD27	PC7	Beckman	B53318	081124_01	1A4CD27	Human
	CD4	APC	Beckman	737660	081124_01	13B8.2	Human
	CD8	A700	Beckman	B53328	081124_01	B9.11	Human
	CD3	APC-A750	Beckman	A07748	081124_01	UCHT1	Human
	CD57	PB	Beckman	B53328	081124_01	NC1	
	CD45	Krome Orange	Beckman	A96416	081124_01	J33	Human

Supplementary Figure 1

Immune subsets in FL and CLL at different timepoints after COVID-19 vaccination

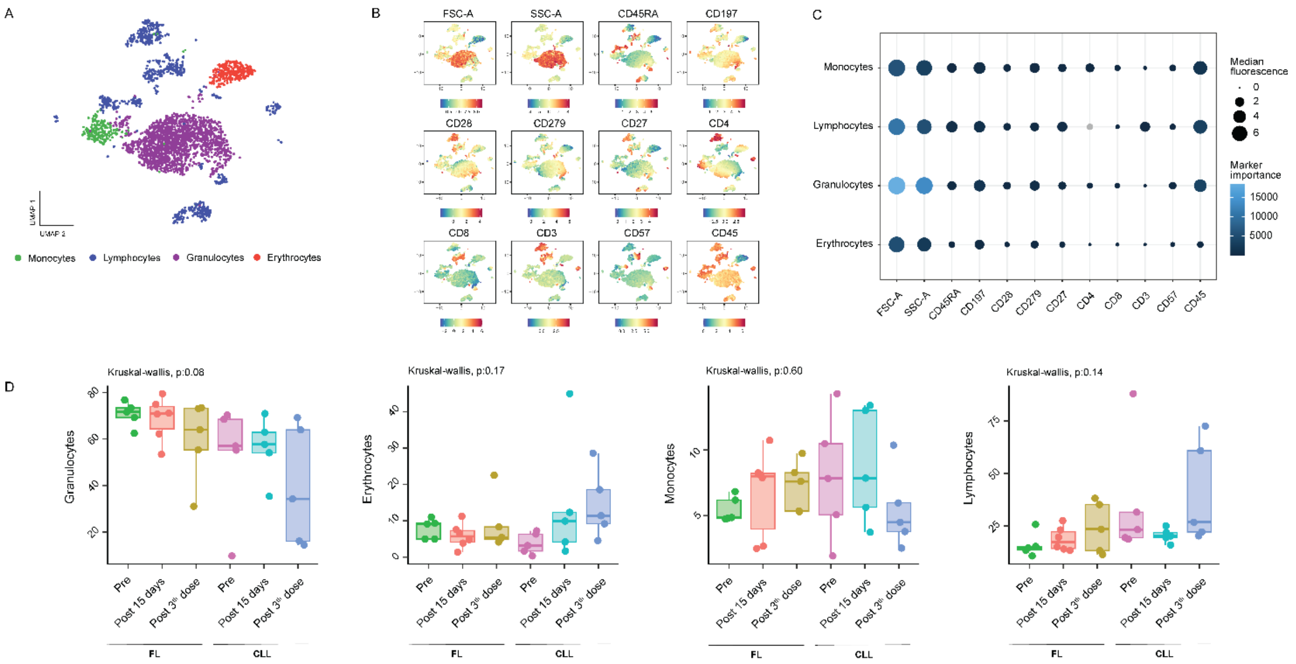

(A) Uniform manifold approximation and projection (UMAP) of erythrocytes (CD45-, SSClow), granulocytes (CD45+dim, SSChigh), lymphocytes (CD45+, SSClow), and monocytes (CD45+, SSCint) identified via a self-organizing map (SOM). (B) Uniform manifold approximation and projection (UMAP) illustrating the expression of each marker within immune cell populations identified via a self-organizing map (SOM). (C) Dot plot with median fluorescence values for each marker (x-axis) across all clusters (y-axis) within immune cell populations identified via a self-organizing map (SOM). (D) Boxplots comparing the proportions of granulocytes, erythrocytes, monocytes, and lymphocytes between FL and CLL patients at baseline, 15 days after Dose 1 (T1), and 30 days after Dose 3 (T3).

Abbreviations: CLL, chronic lymphatic leukemia; FL.

Supplementary Figure 2

Monocyte subsets in FL and CLL at different timepoints after COVID-19 vaccination

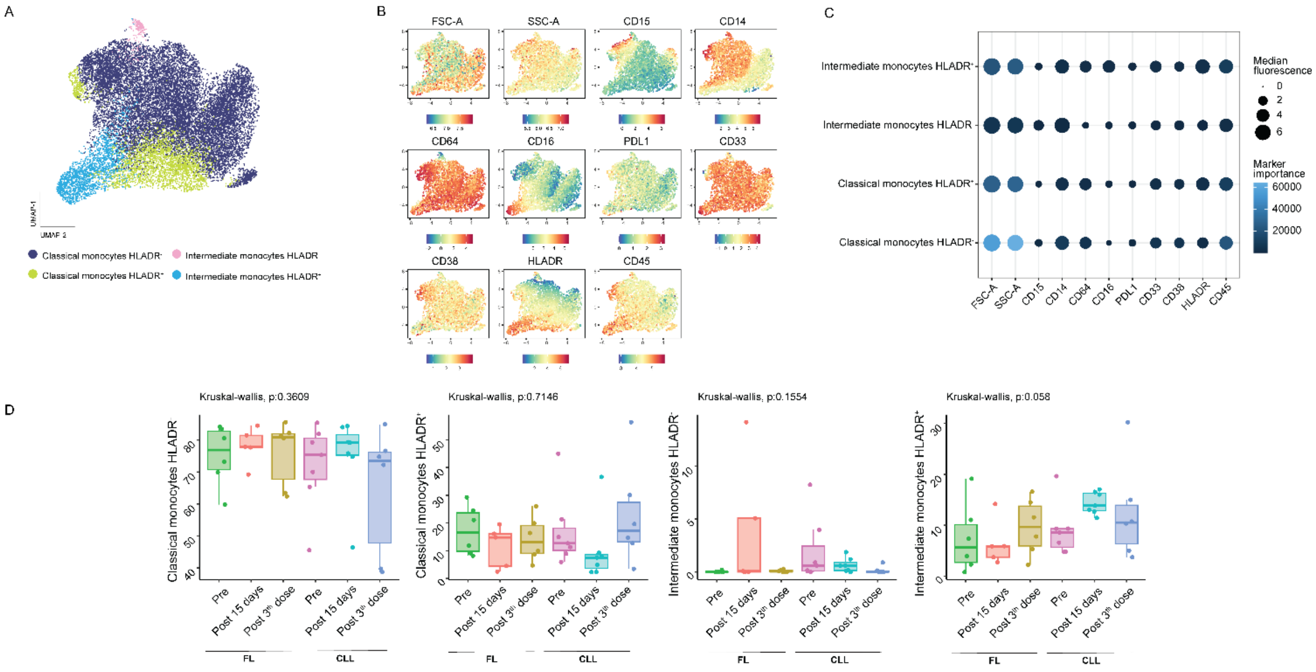

(A) Uniform manifold approximation and projection (UMAP) of monocyte subsets identified via a self-organizing map (SOM). (B) Uniform manifold approximation and projection (UMAP) illustrating the expression of each marker within monocyte populations identified via a self-organizing map (SOM). (C) Dot plot with median fluorescence values for each marker (x-axis) across all clusters (y-axis) within monocyte populations identified via self-organizing map (SOM). (D) Boxplots representing the proportions of monocyte subsets in FL and CLL patients at baseline, 15 days after Dose 1 (T1), and 30 days after Dose 3 (T3).

Abbreviations: CLL, chronic lymphatic leukemia; FL.

Supplementary Figure 3

Neutrophil subsets in FL and CLL at different timepoints after COVID-19 vaccination

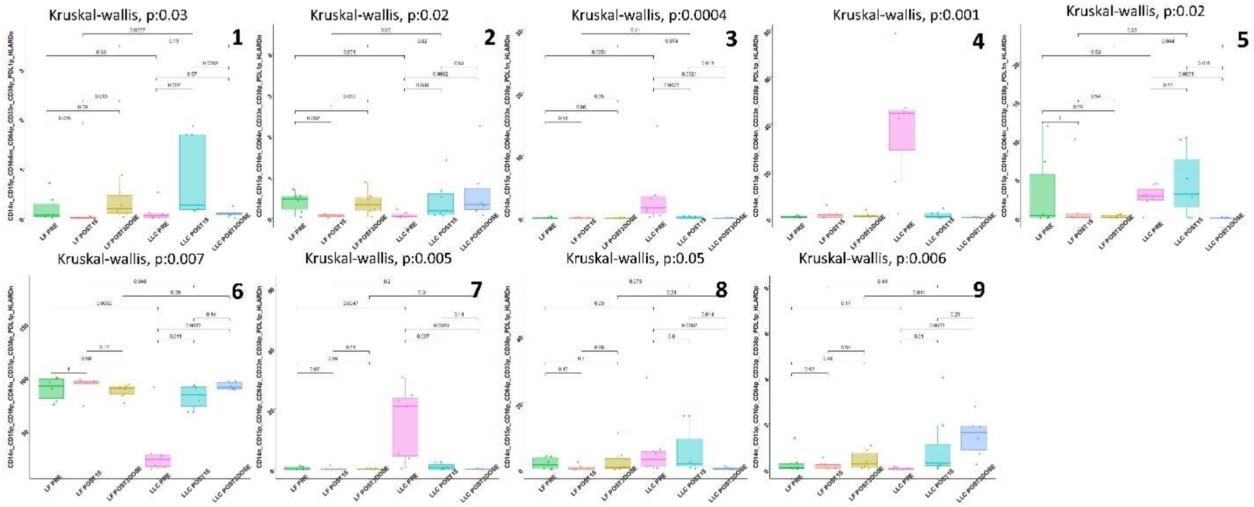

Myeloid-1: $CD14^{-}CD15^{+}CD16^{dim}CD64^{+}CD33^{-}CD38^{+}PDL1^{+}HLA\text{-}DR^{-}$
Myeloid-2: $CD14^{-}CD15^{+}CD16^{-}CD64^{-}CD33^{-}CD38^{+}PDL1^{+}HLA\text{-}DR^{-}$
Myeloid-3: $CD14^{-}CD15^{+}CD16^{+}CD64^{-}CD33^{-}CD38^{-}PDL1^{-}HLA\text{-}DR^{-}$
Myeloid-4: $CD14^{-}CD15^{+}CD16^{-}CD64^{-}CD33^{-}CD38^{+}PDL1^{-}HLA\text{-}DR^{-}$
Myeloid-5: $CD14^{-}CD15^{+}CD16^{+}CD64^{-}CD33^{+}CD38^{+}PDL1^{-}HLA\text{-}DR^{-}$
Myeloid-6: $CD14^{-}CD15^{+}CD16^{+}CD64^{-}CD33^{+}CD38^{+}PDL1^{+}HLA\text{-}DR^{-}$
Myeloid-7: $CD14^{-}CD15^{+}CD16^{+}CD64^{+}CD33^{-}CD38^{+}PDL1^{+}HLA\text{-}DR^{-}$
Myeloid-8: $CD14^{-}CD15^{+}CD16^{+}CD64^{+}CD33^{+}CD38^{+}PDL1^{-}HLA\text{-}DR^{-}$
Myeloid-9: $CD14^{-}CD15^{+}CD16^{+}CD64^{+}CD33^{+}CD38^{+}PDL1^{-}HLA\text{-}DR^{+}$

Boxplots representing the proportions of neutrophil subsets in FL and CLL patients at baseline, 15 days after Dose 1 (T1), and 30 days after Dose 3 (T3).
Abbreviations: CLL, chronic lymphatic leukemia; FL.

Supplementary Figure 4

Evaluation of alpha diversity based on total amount of IgG before and after COVID-19 vaccination in CLL patients

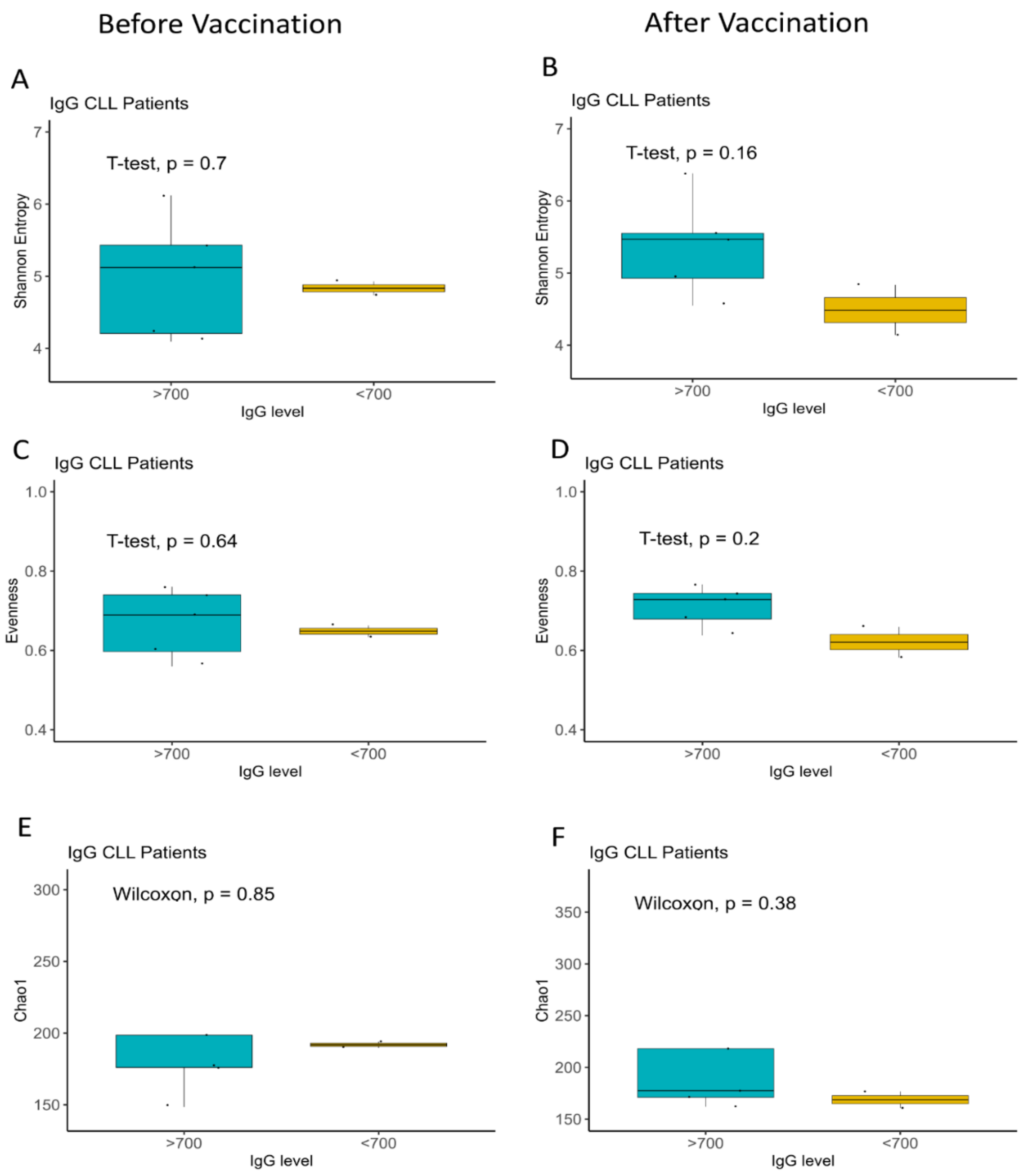

Comparison between levels of IgG (normal >700 and low < 700) and alpha diversity index in CLL patients before and after SARS-CoV-2 vaccination. (A and B) correlation between normal and low level of IgG and Shannon Entropy index before and after COVID-19 vaccination; (C and D) correlation between normal and low level of IgG and Eveness index before and after COVID-19 vaccination; (E and F) correlation between normal and low level of IgG and Chao1 index before and after COVID-19 vaccination. The differences between groups have been calculated employing t-test in case of normal distribution and with Wilcoxon test in case of not-normal distribution. The distribution normality was calculated using Shapiro-wilk test.

Supplementary Figure 5

Bacterial genera abundance percentages in CLL patients before and after vaccination

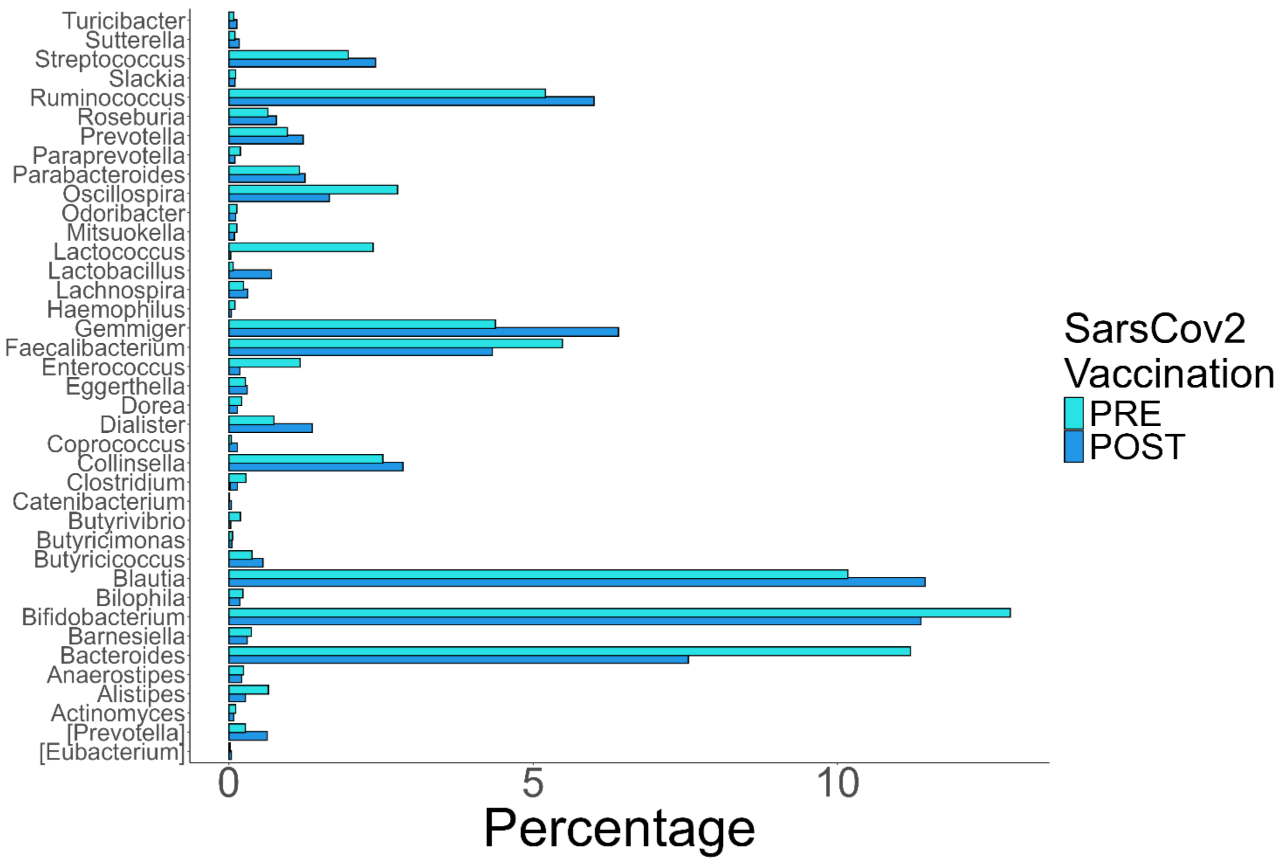

At the genus level, the percentages of major microbial populations that increase or decrease before or after vaccination in CLL patients

Supplementary Figure 6

Correlation between specific neutrophils subsets affected by vaccination and genera abundance in CLL patients

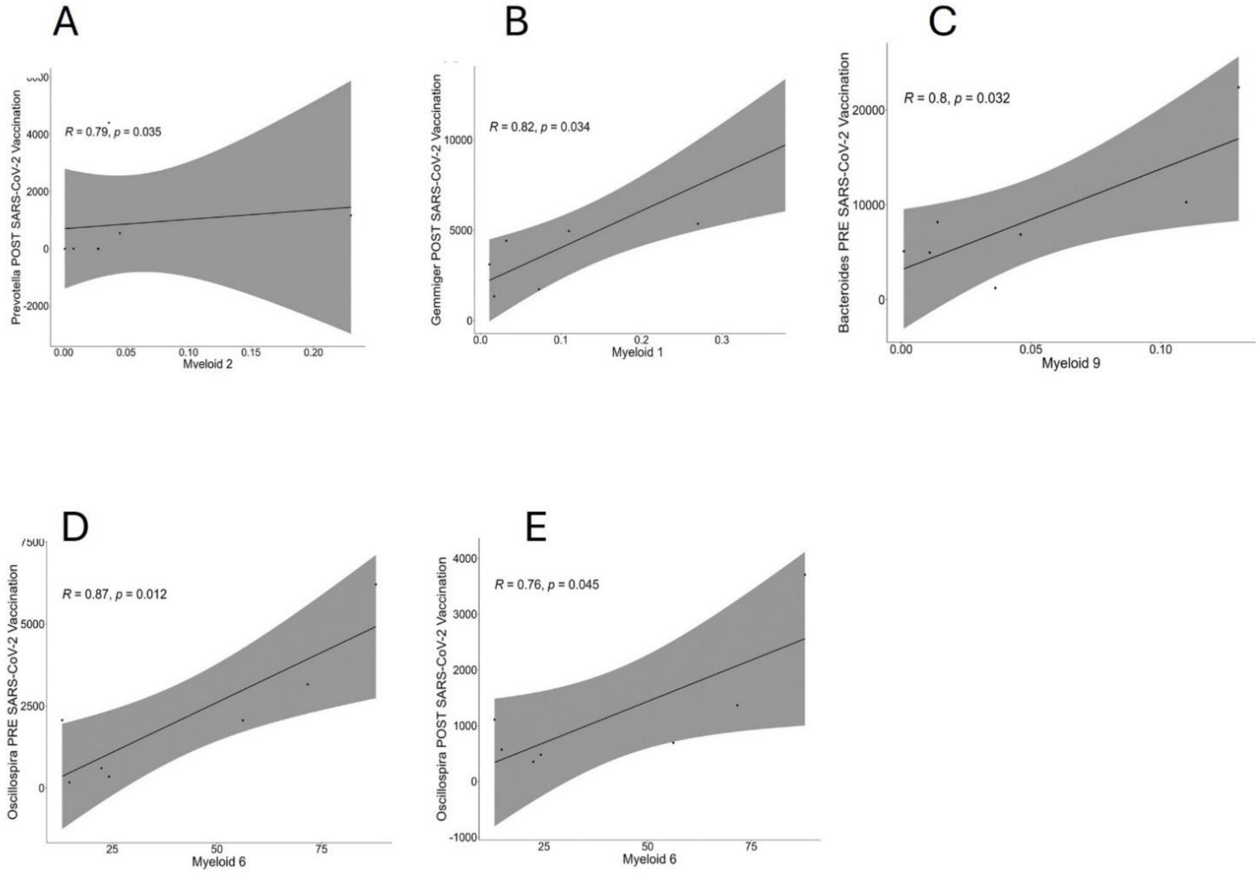

Myeloid-1: $CD14^-CD15^+CD16^{dim}CD64^+CD33^-CD38^+PDL1^+HLA\text{-}DR^-$

Myeloid-2: $CD14^-CD15^+CD16^-CD64^-CD33^-CD38^+PDL1^+HLA\text{-}DR^-$

Myeloid-6: $CD14^-CD15^+CD16^+CD64^-CD33^+CD38^+PDL1^+HLA\text{-}DR^-$

Myeloid-9: $CD14^-CD15^+CD16^+CD64^+CD33^+CD38^+PDL1^+HLA\text{-}DR^+$

Significative correlations between Myeloid population and genera absolute abundance in CLL patients. (A) positive correlations between *Prevotella* and Myeloid 2 after vaccination; (B) *Gemmiger* positive correlation respectively with Myeloid 1 in post- SARS-CoV-2 vaccination; (C) positive correlation between *Bacteroides*; (D and E), positive correlations between *Oscillospira* and Myeloid 6 both before after SARS-CoV-2 Vaccination.

Supplementary Figure 7

Bacterial genera abundance percentages in FL patients before and after vaccination

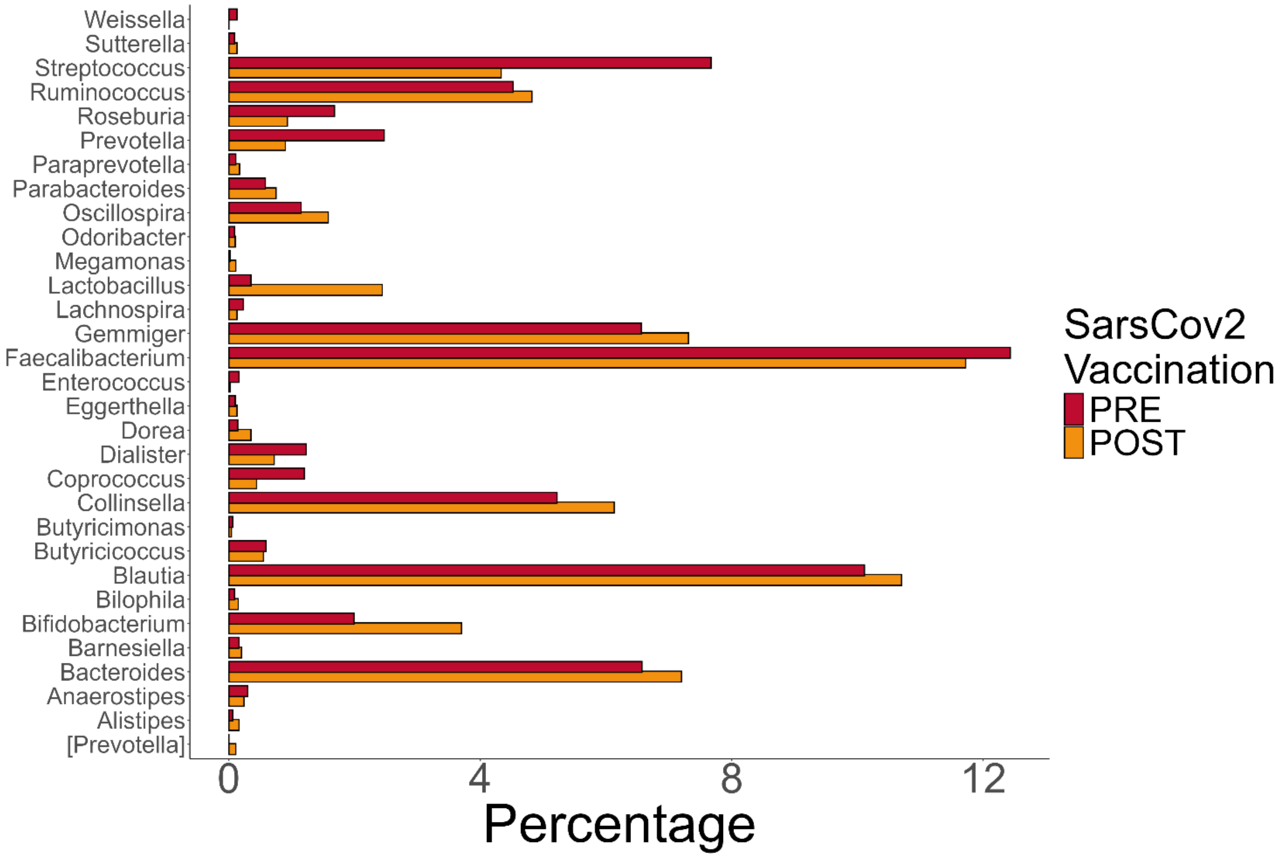

At the genus level, the percentages of major microbial populations that increase or decrease before or after vaccination in FL patients

Supplementary Figure 8

Evaluation of alpha diversity based on total amount of IgG before and after COVID-19 vaccination in FL patients

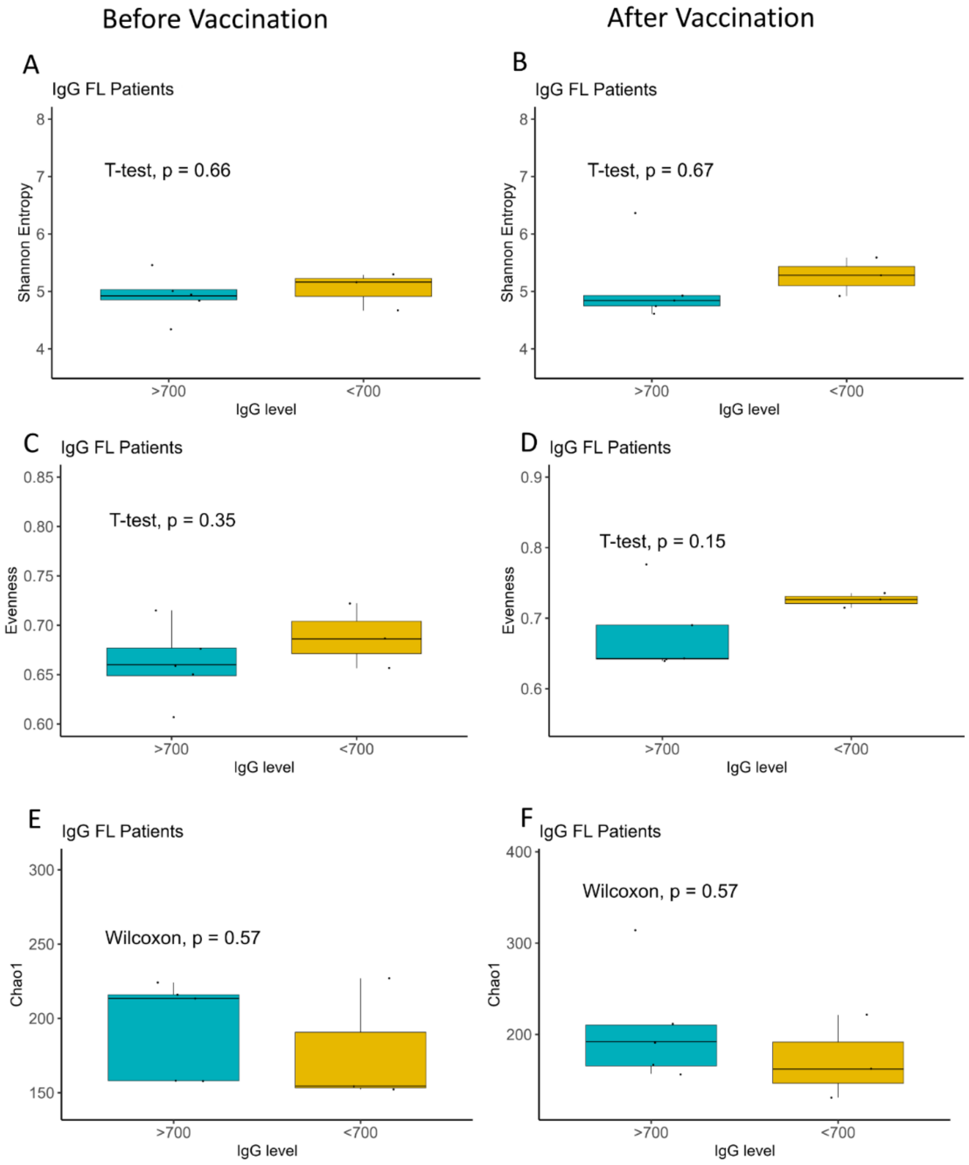

Comparison between levels of IgG (normal >700 and low < 700) and alpha diversity index in FL patients before and after SARS-CoV-2 vaccination. (A and B) correlation between normal and low level of IgG and Shannon Entropy index before and after COVID-19 vaccination; (C and D) correlation between normal and low level of IgG and Eveness index before and after COVID-19 vaccination; (E and F) correlation between normal and low level of IgG and Chao1 index before and after COVID-19 vaccination. The differences between groups have been calculated employing t-test in case of normal distribution and with Wilcoxon test in case of not-normal distribution. The distribution normality was calculated using Shapiro-wilk test.

Supplementary Figure 9

Correlations between alpha-diversity indices and frequence of myeloid subsets in FL patients

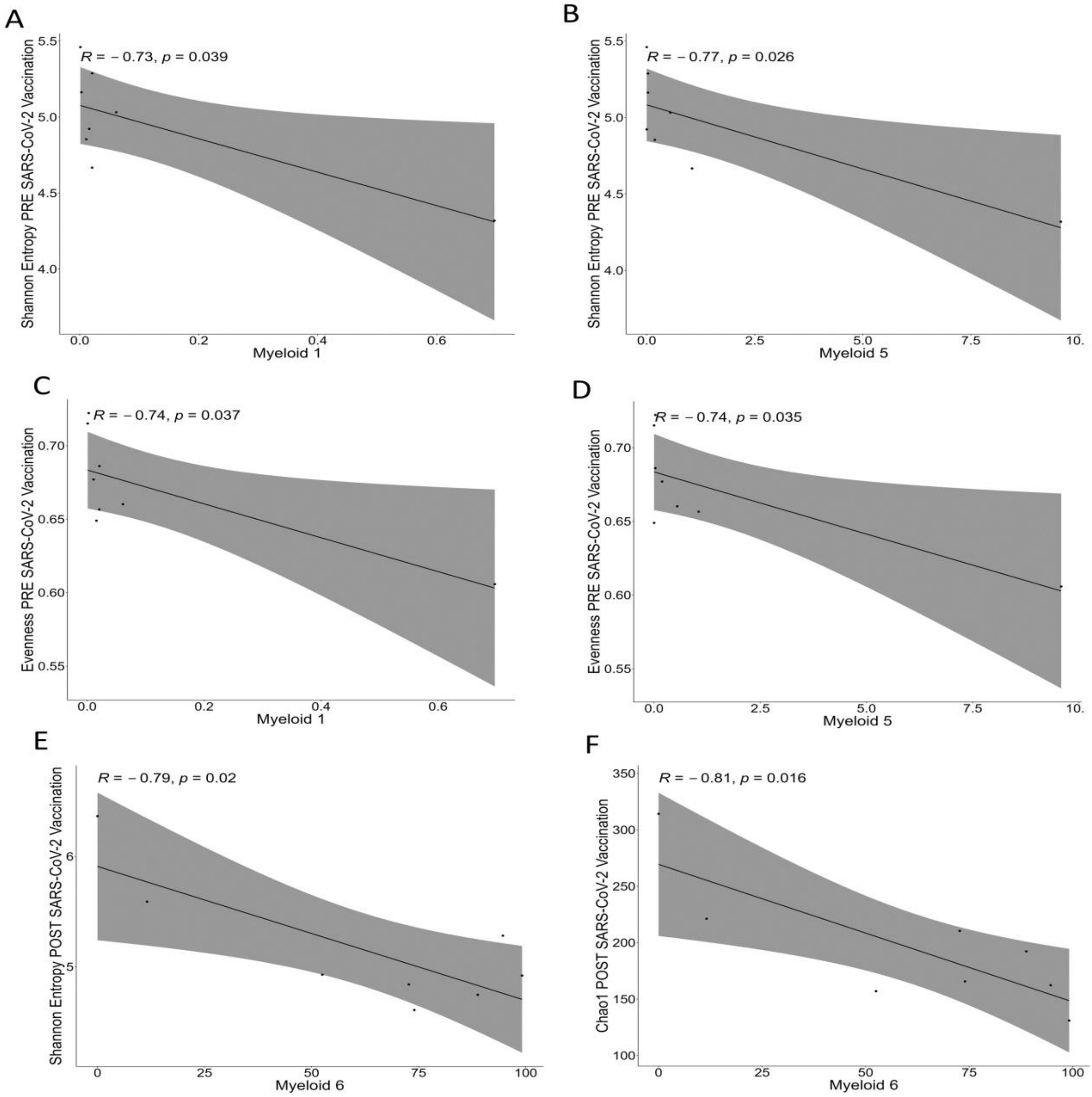

Significative correlations between Myeloid population and alpha diversity index in FL patients. (A and B) negative correlation between Shannon Entropy PRE SARS-CoV-2 Vaccination and respectively Myeloid 1 and Myeloid 5. (C and D) show Evenness PRE SARS-CoV-2 negative correlation with Myeloid 1 and Myeloid 5. (E and F) expose negative correlation of Myeloid 6 respectively with Shannon entropy and Chao1 after SARS-CoV-2 Vaccination. Correlations have been calculated with Pearson or Spearman correlation respectively for normal and not-normal data distribution.

Supplementary Figure 10

Correlation between specific neutrophils subsets affected by vaccination and genera abundance in FL patients

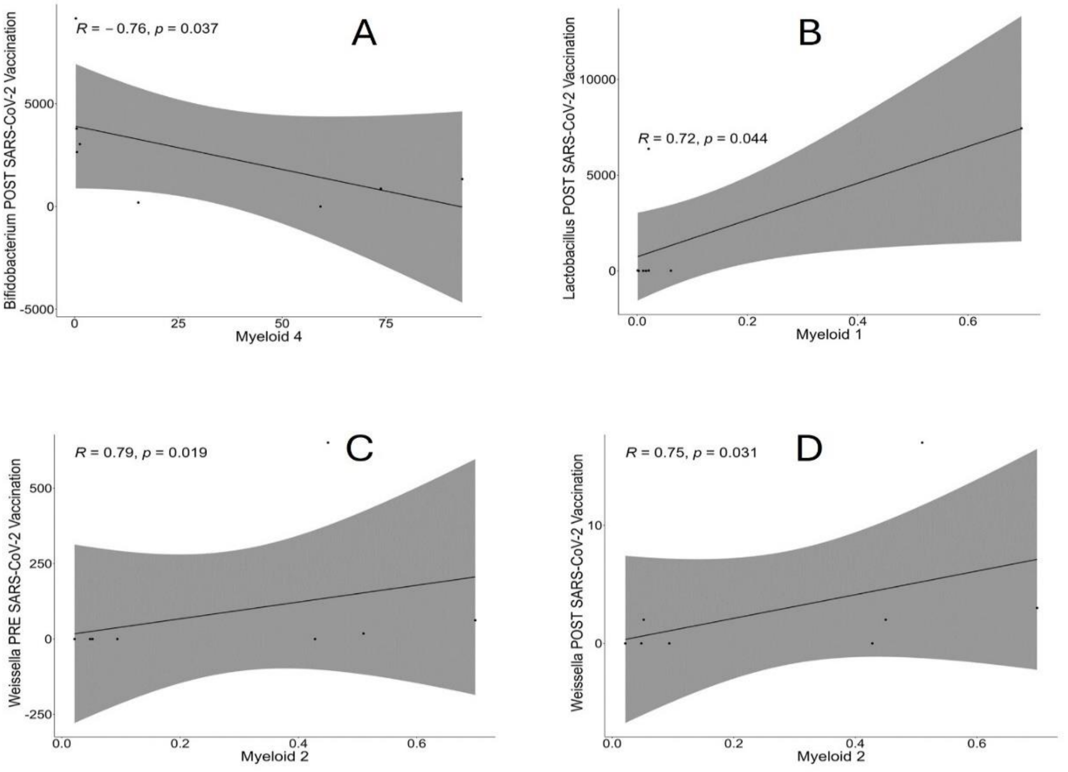

Myeloid-1: $CD14^{-}CD15^{+}CD16^{dim}CD64^{+}CD33^{-}CD38^{+}PDL1^{+}HLA\text{-}DR^{-}$
Myeloid-2: $CD14^{-}CD15^{+}CD16^{-}CD64^{-}CD33^{-}CD38^{+}PDL1^{+}HLA\text{-}DR^{-}$
Myeloid-4: $CD14^{-}CD15^{+}CD16^{-}CD64^{-}CD33^{-}CD38^{+}PDL1^{-}HLA\text{-}DR^{-}$

Significative correlations between Myeloid population and genera absolute abundance in FL patients. (A) negative correlation between *Bifidobacterium* and Myeloid 4 in POST SARS-CoV-2 vaccination; (B) positive correlations between *Lactobacillus* and Myeloid 1, after SARS-CoV-2 vaccination; (C and D) positive correlations between *Weissella* and Myeloid 2 both before and after SARS-CoV-2 Vaccination. Correlations have been calculated with Pearson or Spearman correlation respectively for normal and not-normal data distribution. Correlations have been considered significative with a p-value < 0.05.
